# AAV9-mediated targeting of natural antisense transcript as a novel treatment for Dravet syndrome

**DOI:** 10.1016/j.omtn.2026.102942

**Published:** 2026-04-30

**Authors:** Juan Antinao Diaz, Ellie M. Chilcott, Amanda Almacellas Barbanoj, Anna Keegan, Sonam Gurung, Valda Pauzuolyte, Zak Waddington, Maria Kyriacou, Amy McTague, J Helen Cross, Stephanie Schorge, Gabriele Lignani, Simon N. Waddington, Rajvinder Karda

**Affiliations:** 1 EGA Institute for Women’s Health, University College London, London WC1E 6HX, UK; 2 Research Department of Epilepsy, UCL Queen Square Institute of Neurology, London, WC1N 3BG, UK; 3 UCL Great Ormond Street Institute of Child Health, University College London, London WC1N 1EH, UK; 4 Department of Neurology, Great Ormond Street Hospital, London WC1N 3JH, UK; 5 Division of Biosciences, Medical Science Building, University College London, London WC1E 6BT, UK

**Keywords:** MT, delivery strategies, Dravet syndrome, SMEI, gene therapy, AAV, Na_V_1.1, natural antisense transcript, pre-clinical

## Abstract

Dravet syndrome (DS) is a severe childhood developmental and epileptic encephalopathy. Symptoms usually manifest in the first year of life and include prolonged severe seizures, developmental delay, severe intellectual disability, and increased mortality. Approximately, 90% of patients carry a heterozygous loss-of-function mutation in *SCN1A*, encoding a voltage-gated sodium ion channel, Na_V_1.1. Na_V_1.1 is expressed in the brain and at a lower level, in the heart. Previous studies have identified a long non-coding RNA (lncRNA), which specifically downregulates *SCN1A* expression. This natural antisense transcript (NAT) can be modulated by AntagoNATs, small synthetic oligonucleotides. AntagoNATs have shown to improve seizure frequency in DS mice after repeated administration. Here, we have developed new AntagoNATs and incorporated these into an adeno-associated virus serotype 9 (AAV9) gene therapy vector.

We administered two new AAV9-AntagoNAT-H and AntagoNAT-K vectors to newborn *Scn1a*^*+/−*^ mice via intracerebroventricular (i.c.v.) and intravenous (i.v.) injection to deliver vector to the brain and heart. AAV9-AntagoNAT-H significantly increased survival, decreased the frequency of febrile and spontaneous seizures. In this proof-of-concept study, we have demonstrated for the first time the delivery of AntagoNAT via an AAV9 vector. Thus, offering the possibility of a one-time treatment for DS patients.

## Graphical abstract

**Figure:**
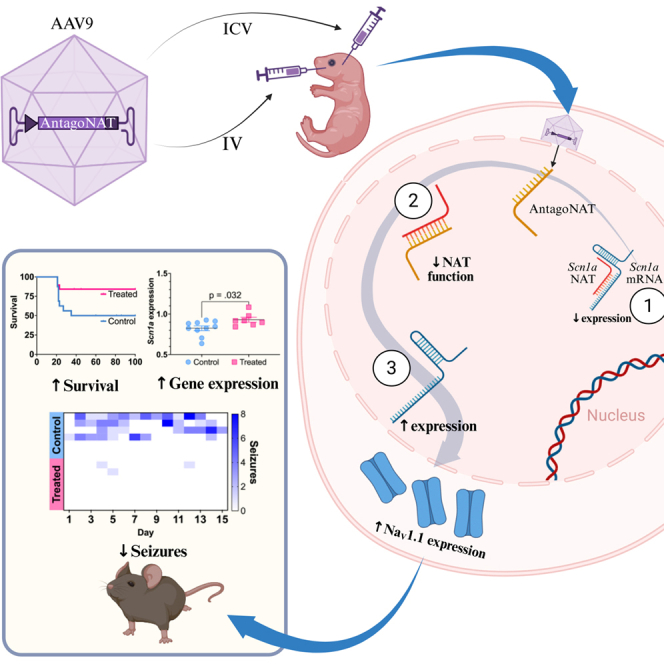

## Teaser

Karda and colleagues showed a novel approach to deliver oligonucleotides using a modified virus, which they tested in a mouse model of genetic epilepsy known as Dravet syndrome. The treatment reduced seizures and improved survival, showing that this approach could provide a one-time treatment for Dravet syndrome patients.

## Introduction

Dravet syndrome (DS) is a severe early-onset genetic epilepsy that manifests in the first year of life, with an incidence of 1 in 15,400 to 1 in 40,900 live births worldwide.^1^^,^^2^ DS patients exhibit frequent prolonged febrile and afebrile seizures, generalized tonic, or hemiclonic seizures, status epilepticus events, with cognitive decline, developmental delay, ataxia, and many other comorbidities.^3^^,^^4^^,^^5^ Unfortunately, DS patients have an increased mortality rate, including sudden unexpected death in epilepsy (SUDEP).^5^

More than 90% of DS patients harbor a heterozygous loss-of-function mutation in the *SCN1A* gene, which encodes the alpha subunit of the voltage-gated ion channel, Na_V_1.1.^1^ In the human brain, Na_V_1.1 expression has been identified in the hippocampus, dentate gyrus, CA3 and CA2, layer V/VI of the cortex, granular layer, molecular layer, deep nuclei and Purkinje cells of the cerebellum.^6^ Animal studies have also identified Na_V_1.1 expression in the hippocampus,^7^ thalamus,^8^ deep cerebellar nuclei,^9^ and the spinal cord,^10^ specifically in inhibitory interneurons,^7^ Purkinje neurons,^11^ and CA1 pyramidal cells.^12^ Na_V_1.1 has been detected in the human heart and studies have investigated altered electrical cardiac function in DS patients and mouse model, suggesting a possible contribution to SUDEP.^13^^,^^14^^,^^15^^,^^16^

Multiple models of DS have been established to study disease mechanisms, which have demonstrated spontaneous seizures, febrile seizures, SUDEP, cognitive deficits, and other comorbidities.^1^ Many of these models have shown a notable dependence on genetic background.^17^ Yu et al.^7^ developed a heterozygous loss of function DS model (*Scn1a*^*+/−*^), where *Scn1a* exon 25 was disrupted. The study revealed, *Scn1a*^*+/−*^ mice on a C57BL/6 J strain resulted in a more severe disease phenotype than on a 129/SvJ background. They also developed a mix DS model 129/SvJ:C57BL/6 J, demonstrating that Na_V_1.1 loss of function in inhibitory interneurons in the hippocampus resulted in spontaneous seizures and increased mortality.^7^ Cheah et al.^18^ provided further evidence of the importance of GABAergic interneurons in DS by selectively deleting *Scn1a* in forebrain GABAergic neurons, which also resulted in tonic-clonic seizures, febrile seizures, and premature death. Another *Scn1a*^*+/−*^ model was developed by deleting the first coding exon of *Scn1a* in a mixed 129/SvJ:C57BL/6 J background strain. The first generation of *Scn1a*^*+/−*^ mice demonstrated seizures and premature death.^19^ Further studies on this *Scn1a*^*+/−*^ model have shown a reduction in sodium currents from hippocampal inhibitory GABAergic interneurons. Interestingly, they also noted elevated sodium currents in excitatory pyramidal neurons of the hippocampus.^17^ In addition, studies have also demonstrated altered excitatory CA1 pyramidal neuronal networks during early stages of development in the *Scn1a*^*+/−*^ model.^20^ Together, these studies illustrate the involvement of both inhibitory and excitatory neuronal networks in DS.

Other organs such as the heart have also shown involvement in DS. DS patients demonstrate autonomic symptoms,^13^ in some cases resulting in reduced heart rate variability, which may act as a potential biomarker for SUDEP risk.^21^ DS mouse model have also showed an increase of sodium currents in ventricular myocytes resulting in altered electrical cardiac function.^14^

Overall, the DS mouse model studies have demonstrated that the loss of *Scn1a* in multiple regions of the brain and the association of the heart contributes to disease phenotype. Therefore, there is a need to develop a widespread genetic therapy approach to restore function of *SCN1A* in multiple neuronal cells and the heart.

Current anti-seizure medications such as valproate, stiripentol, and clobazam^22^ are often ineffective in reducing seizure frequency and other comorbidities associated with DS. Therefore, there is a great need for alternative therapies. Gene supplementation therapy using adeno-associated viral (AAV) vectors have demonstrated tremendous clinical success for severe early-onset neurological disorders.^23^^,^^24^ However, the *SCN1A* gene (6kb), exceeds the packaging capacity of AAV (∼4.7kb).^25^ Therefore, gene supplementation using a single AAV is not feasible for treating *SCN1A*-related DS. A dual AAV approach has been employed delivering *SCN1A* in two halves.^26^ A gene supplementation approach using an adenoviral vector has been developed for DS, as it has a larger packaging capacity than AAV.^27^ Numerous other genetic therapies for DS have been developed, which aim to upregulate *SCN1A* expression and some of these are currently being evaluated in clinical trials. Stoke Therapeutics has developed an antisense oligonucleotide (ASO) therapy, that aims to increase the synthesis of productive *SCN1A* mRNA by preventing the inclusion of nonproductive exon during mRNA splicing.^28^ This treatment has been investigated in phase 1/2a clinical trials, where the ASO is delivered via repeated intrathecal delivery to DS patients.^29^ Early clinical data have shown improvements in overall clinical status.^30^ Encoded Therapeutics has also developed an adeno-associated virus serotype 9 (AAV9)-based therapy to deliver a transcription factor to upregulate *SCN1A*, specifically in GABAergic inhibitory interneurons,^31^ which is currently in phase 1/2 clinical trial.^32^

AntagoNATs are small synthetic oligonucleotides that modulate RNA function, in this case by targeting the natural antisense transcript (NAT) of *SCN1A*, thus inhibiting the NAT and increasing *SCN1A* mRNA and consequently, Na_V_1.1 protein production.^33^ A reduction of seizure phenotype after four repeated intrathecal administration (once per week) of AntagoNATs to 7-week-old DS knockin mice has been demonstrated.^33^

We sought to improve this approach by designing new AntagoNAT sequences and incorporating them into a clinically relevant AAV vector. Thus, providing a one-off treatment to a clinically relevant DS mouse model.^28^ AAV9 serotype was selected for this study as this has been used extensively in many preclinical and clinical studies for central nervous system (CNS) diseases.^24^^,^^31^ Furthermore, we aimed to target the brain and the heart with our therapy, as studies have shown Na_V_1.1 to be expressed in these areas.^7^^,^^16^^,^^17^^,^^18^^,^^20^

The *in vitro* analysis identified candidate AntagoNAT sequences K and H, which showed a higher increase in endogenous *Scn1a* expression when compared to previously published AntagoNAT sequence.^33^ We tested these with a heterozygous *Scn1a*^*+/−*^ Dravet mouse model, which shows spontaneous seizures, febrile seizures, and increased mortality.^17^^,^^28^ AAV9-AntagoNAT-K and AAV9-AntagoNAT-H were administered to newborn *Scn1a*^*+/−*^ DS mice via intracerebroventricular (i.c.v.) alone, and i.c.v. and intravenous (i.v.) delivery. The results revealed a significant increase in survival (from 50% to 84%), significant reduction in febrile seizures susceptibility (73% down to 7.7%) and a reduction in average daily seizure frequency (1.12 down to 0.03) in mice treated with AAV9-AntagoNAT-H via i.c.v. and i.v. delivery. Furthermore AAV9-AntagoNAT-H delivered to older *Scn1a*^*+/−*^ mice by combination therapy led to an increase cortical endogenous *Scn1a*.

## Results

### Development of new AntagoNAT sequences

**Figure 1:**
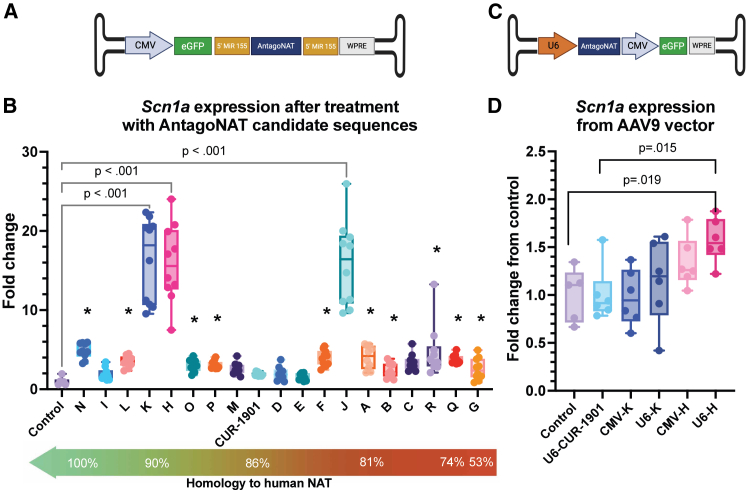
*In vitro* assessment of new AntagoNAT sequences (A) Schematic of AAV-CMV-eGFP-miR-155-AntagoNAT-miR-155-WPRE plasmid. (B) Transient transfection revealed 13 sequences to significantly increase *Scn1a* expression compared to the control cells (∗*p* < 0.001). Candidates K, H, and J showed the highest increase in expression (16-fold compared to control). One-way ANOVA with multiple comparison correction using Two-stage linear step-up procedure of Benjamini, Krieger, and Yekutieli. *n* = 9/10 for AntagoNAT sequences, *n* = 6 for control (3 technical replicates each). (C) Schematic of AAV-U6-AntagoNAT-CMV-eGFP-WPRE plasmid. (D) *In vitro* transduction of AAV9 vectors (*n* = 5/6 replicates of each group, 3 technical replicates each) using either the U6 or the CMV promoter, represented in a Boxplot. One-way ANOVA, Dunnett’s multiple comparison performed.

To investigate whether alternative AntagoNAT sequences provided superior survival and protection against seizures, compared to the published sequence (CUR-1901),^33^ we designed 18 new AntagoNAT sequences. We designed new AntagoNAT sequences based on the secondary *Scn1a* NAT structure, targeting regions not previously studied by Hsiao et al.,^33^ aiming to achieve improved efficacy. We incorporated these into an AAV plasmid backbone containing an RNA polymerase II CMV (Cytomegalovirus) promoter, enhanced green fluorescent protein (eGFP), and the AntagoNAT sequences, which were flanked by miR-155 sequences (Figure 1A).^34^ miR-155 sequences are short hairpin-looped structures, which allow stable transcription of small RNA sequences (RNA-seq) by RNA polymerase II promoters.^34^

We transfected the AAV backbone constructs into differentiated Neuro2a (N2a) cells. Thirteen of the AntagoNAT sequences significantly increased endogenous *Scn1a* expression compared to AAV-CMV-GFP control, ranging between 3.4-fold (1.00) for candidate C, to 16-fold (4.90) for candidate H (Figure 1B). AntagoNAT sequences H, J, and K showed the highest increase in *Scn1a* expression (∼16-fold increase each; *p* < 0.001), with K and H being the most homologous to the human NAT sequence (90%). In this study, we aimed to obtain a significant increase of endogenous *Scn1a* expression *in vivo* coupled with a lower vector dose of administration and therefore, AntagoNAT-K and AntagoNAT-H were chosen for the *in vivo* preclinical study. CUR-1901,^33^ showed an increase in endogenous *Scn1a* expression to 1.9 (0.23)-fold compared to control, when delivered from our plasmid construct (Figure 1B).

Before we progressed to the *in vivo* study, we compared the CMV promoter versus a U6 RNA polymerase III promoter (Figure 1C). In general, RNA polymerase III promoters are suitable to drive small RNA-seq, which do not require protein translation.^35^ AAV9 vectors were produced with both promoters and used to transduce N2a cells. The U6 promoter yielded a significant increase in endogenous *Scn1a* expression with AntagoNAT-H, when compared to control and CUR-1901 groups (Figure 1D). AntagoNAT-K also showed an increase in endogenous *Scn1a* expression compared to control (Figure 1D). As the AAV9-U6 constructs showed a better performance *in vitro* compared to the original CMV, we therefore proceeded with the AAV9-U6-AntagoNAT-H and AntagoNAT-K vectors for the *in vivo* study.

### Neonatal delivery of AAV9-AntagoNAT-H reduces SUDEP and seizure phenotype in a DS mouse model

To test the efficacy of our AntagoNAT sequences, we produced an AAV9 vector to deliver AntagoNAT-H and AntagoNAT-K, and we used AntagoNAT-CUR-1901 for comparison. We tested the biodistribution, efficacy, and safety of our AAV9-AntagoNAT therapy vectors and AAV9-CUR-1901 in the *Scn1a*^+/−^ and *Scn1a*^+/+^ mice.^17^

**Figure 2:**
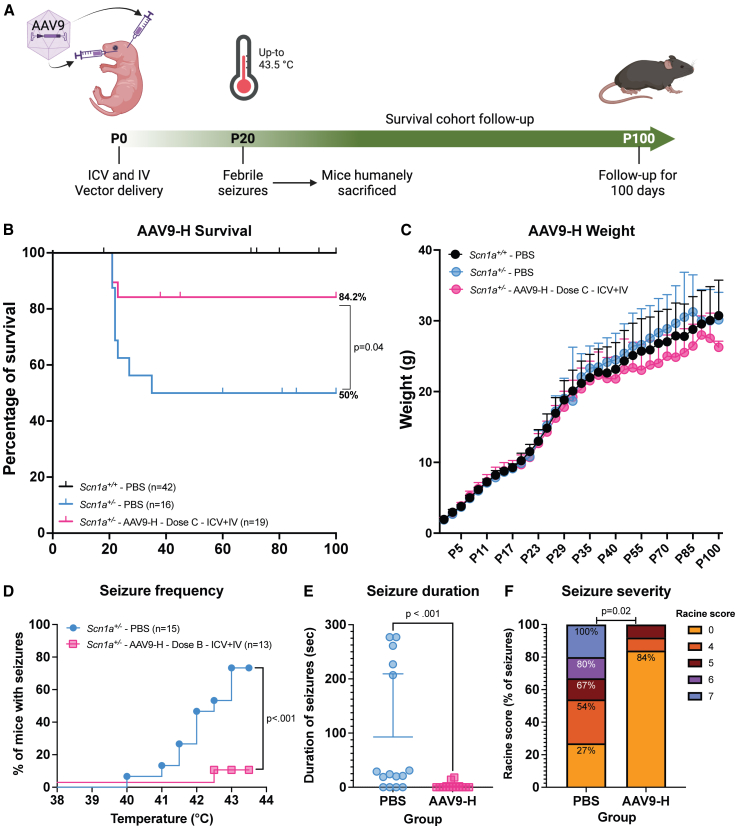
Neonatal AAV9-AntagoNAT-H gene therapy increases survival and reduces febrile seizures in DS mice *Scn1a*^*+/−*^ mice received either AAV9-AntagoNAT-H or PBS via i.c.v. and i.v. PBS was also administered to wild-type mice (*Scn1a*^*+/+*^). (A) Experiment timeline. (B) Survival of *Scn1a*^*+/−*^ DS mice treated with AAV9-AntagoNAT-H (*p* = 0.04), shown in percentage of survival. Log-rank (Mantel-Cox) test. (C) Weight curves, data presented as mean, with SD error bars. Two-way ANOVA with Dunnett’s multiple comparisons test. (D) Febrile seizure temperature threshold assessment, showing a significant reduction with AAV9-AntagoNAT-H treated group (*p* < 0.001). Log-rank (Mantel-Cox) test. (E) Duration of febrile seizures reduced in AAV9-AntagoNAT-H group (*p* < 0.001). Man-Whitney test. (F) The Racine score classification of febrile seizures observed in the experimental mice. A significant reduction in seizure severity was observed in AAV9-AntagoNAT-H group (*p* = 0.02). Fisher's exact test.

We administered the AAV9-AntagoNAT-H, AAV9-AntagoNAT-K, and AAV9-CUR-1901 to newborn *Scn1a*^*+/−*^ and *Scn1a*^+/+^ mice via bilateral i.c.v. (dose A: 1 × 10^11^ vg/mouse, dose B: 1 × 10^10^ vg/mouse; Table S1) or a combination therapy of i.c.v. and i.v. (dose C: 3.5 × 10^10^ vg/mouse; Table S1) delivery to target the brain and the heart. The study was fully blinded and randomized (Figure 2A).

AAV9-AntagoNAT-H via i.c.v. and i.v. (dose C) significantly increased survival to 84.2% (*p* = 0.04, Figure 2B) compared to 50% of phoshate-buffered saline (PBS) control *Scn1a*^*+/−*^ mice at P100. No difference in weight was observed (Figure 2C). In contrast, delivery of AAV9-CUR-1901 showed a 77% survival, AAV9-AntagoNAT-K revealed 58% survival, compared to 50% of PBS control *Scn1a*^*+/−*^ mice at P100 (Figures S1A and S1C). Neonatal i.c.v. delivery of dose A resulted in 60% survival for AAV9-AntagoNAT-K, 57% survival for AAV9-CUR-1901, and 12.5% for AAV9-AntagoNAT-H, compared to 50% of PBS control *Scn1a*^*+/−*^ mice at P100 (Figures S1A, S1C, and S1E). No improvement in survival was observed with neonatal i.c.v. delivery of dose B in all treatment groups (Figures S1A, S1C, and S1E). No difference in weight was observed between any of the treatment groups (Figures S1B, S1D, and S1F). AAV9-AntagoNAT-H, AAV9-AntagoNAT-K, and CUR-1901 i.c.v. dose A, induced open-field hyperactivity compared to PBS-treated *Scn1a*^+/+^ controls (Figure S2A). Whereas i.c.v. dose B and combination therapy dose C with all AAV9 vectors showed no hyperactivity phenotype during open-field assessments (Figures S2B and S2C).

We observed a 100% survival and no difference in weight in all *Scn1a*^+/+^ mice treated with AAV9-AntagoNAT-H, AAV9-AntagoNAT-K, and AAV9-CUR-1901 via i.c.v. (dose A and B) or a combination i.c.v. and i.v. therapy (dose C; Figures S3A–S3F). Open-field assessment revealed hyperactivity with AAV9-AntagoNAT-H i.c.v. (dose A) group over development compared to PBS *Scn1a*^+/+^ control group (Figure S4A). Hyperactivity phenotype was only observed at P18 with AAV9-AntagoNAT-H, i.c.v., and i.v. group but was not visible at later time points (Figure S4C). Expression of glial fibrillary acidic protein (GFAP) in astrocytes and CD68 in microglia was assessed in the brain of all *Scn1a*^+/+^ treatment groups and no significant difference was observed compared to *Scn1a*^+/+^ controls (Figures S5A and S5D).

We assessed GFP in the brain and the heart of *Scn1a*^+/+^ mice treated with AAV9-AntagoNAT-H via i.c.v. and combination therapy. We observed widespread GFP expression in the brain (Figure S5E). There was a higher vector copy number (VCN) in the i.c.v. alone (dose A) compared to i.c.v. and i.v. group in both the cortex and heart (Figures S5G and S5I). We observed no upregulation of CD68 (a marker of Kupffer cells and macrophages) in the liver of both i.c.v. and i.v. group (Figure S5J). However, there was significantly greater VCN in the liver of i.c.v. (dose A) group (Figure S5K).

AAV9-AntagoNAT-H via i.c.v. and i.v. (dose C) demonstrated a significant increase in survival, with no behavioral abnormalities when administered to both newborn *Scn1a*^*+/−*^ and *Scn1a*^+/+^ mice, whereas i.c.v. dose A and B showed no effects in survival in *Scn1a*^*+/−*^-treated mice and dose A was associated with hyperactivity. Therefore, we continued the preclinical gene therapy study with this vector and i.c.v. and i.v. combination treatment.

We examined the susceptibility to febrile seizures, as this is a common symptom among DS patients.^36^ By P25, approximately 40% of *Scn1a*^*+/−*^ mice undergo SUDEP, starting at P21 (Figure 2B); therefore, we conducted temperature-induced seizures just before this, at P20. *Scn1a*^*+/−*^ mice, which received i.c.v. and i.v. AAV9-AntagoNAT-H dose C or PBS at P0 were subjected to an increase of temperature from 37°C to 43.5°C, in increments of 0.5°C per min. Only 2 of 13 mice receiving vector exhibited a seizure, whereas 11 out of 15 PBS mice had seizures (*p* < 0.001; Figure 2D, Videos S1 and S2). Furthermore, gene therapy significantly reduced seizure duration (*p* < 0.001; Figure 2E). AAV9-AntagoNAT-H reduced mean Racine score (0.69 (1.70) vs. PBS control 3.9 (2.68); *p* < 0.02) (Figure 2F).

Video S1. Febrile seizure recording from Scn1a+/− mouse

Video S2. Febrile seizure recording from AAV9-H-treated Scn1a+/− mouse

**Figure 3:**
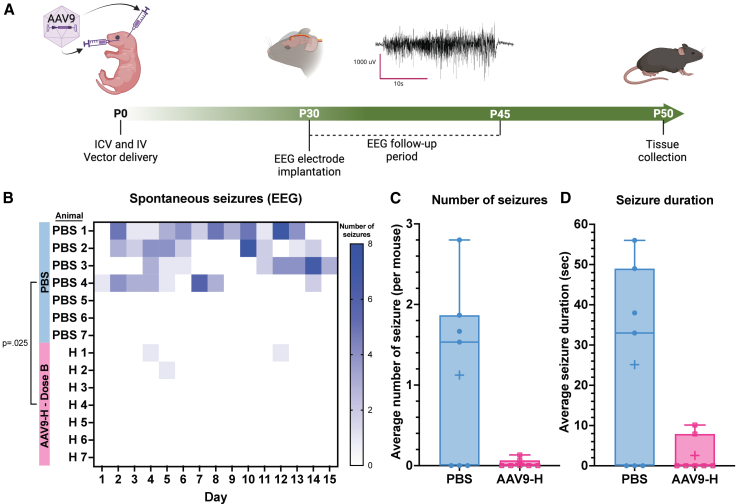
Improved seizure phenotype after neonatal gene transfer of AAV9-AntagoNAT-H (A) Experiment schematic. (B) Heatmap illustrating seizure events per day over 15 days. Significant reduction in seizure events observed in AAV9-AntagoNAT-H group (p=0.025) compared to PBS group. Two-way ANOVA with a post hoc analysis using the Greenhouse-Geisser correction. (C) The average number of seizure events per mouse per day. unpaired Mann-Whitney test. (D) Average seizure duration over the recording period between the AAV9-AntagoNAT-H and PBS control groups. Unpaired Mann-Whitney test.

We measured spontaneous seizure frequency via electroencephalogram (EEG) recordings for 15 days (P30–45, Figure 3A). We observed a significant reduction (*p* = 0.025; Figure 3B) in number of seizures over 15 days after gene therapy compared to PBS. The average daily seizures per mouse after gene therapy were 0.03 (0.05) compared to 1.12 (1.12) for PBS (Figure 3C), We also observed a decrease in seizure duration between treatment groups over 15 days, from 25.14 (24.65) to 2.56 (4.44) s (Figure 3D). Overall, in this neonatal study, we observed a substantial increase in survival and a decrease in seizures by both febrile and EEG seizure recordings.

**Figure 4:**
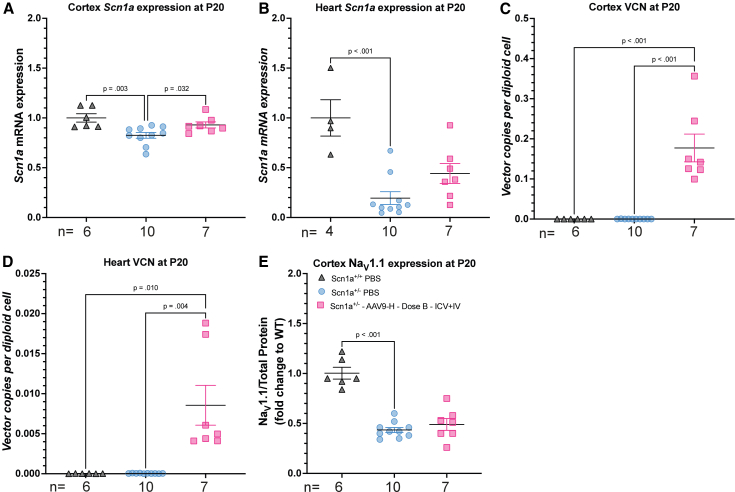
Increase of endogenous *Scn1a* in the cortex of AAV9-AntagoNAT-H-treated *Scn1a*^*+/−*^ DS mice (A) *Scn1a* expression in the cortex, significantly increased with AAV9-AntagoNAT-H (*p* = 0.032) and *Scn1a*^*+/+*^ PBS (*p* = 0.003) compared to PBS group. (B) *Scn1a* expression in the heart showed a signifcant difference between *Scn1a*^*+/+*^ PBS and *Scn1a*^*+/−*^ group (*p* < 0.001) only. (C) Vector copy number analysis in the cortex showed a significant increase in AAV9-AntagoNAT-H group (*p* < 0.001). (D) Vector copy number in the heart only revelaed a signifcant difference between *Scn1a*^*+/+*^ and *Scn1a*^*+/^−^*^ PBS groups (*p* < 0.001). (E) Na_V_1.1 expression in the cortex. One-way ANOVA Holm-Šídák’s multiple comparisons test. For each group, *n* values are indicated in the figure.

A subset of animals from the longitudinal neonatal study (Figure 2B) were collected at P20 and used for molecular assessment. In the cerebral cortex, we observed a significant increase in endogenous *Scn1a* mRNA in AAV9-AntagoNAT-H *Scn1a*^*+/−*^ group, compared to PBS (*p* = 0.036; Figure 4A). In the heart, we observed an increased trend of endogenous *Scn1a* in treated group but did not reach significance compared to PBS controls (Figure 4B). VCN were significantly higher in AAV9-AntagoNAT-H *Scn1a*^*+/−*^ group compared to control groups at P20 (cerebral cortex; *p* < 0.001, heart: *p* = 0.004; Figures 4C and 4D). At P100, VCN was only significant in the cortex but not heart (Figures S6B and S6D). Furthermore, Na_V_1.1 expression in the cerebral cortex for AAV9-AntagoNAT-H *Scn1a*^*+/−*^ group (0.49 [0.16]) was not significant when compared to PBS control group (0.44 [0.08]) (Figure 4E). In addition, *Scn1a* mRNA, VCN, and Na_V_1.1 expression was also assessed for *Scn1a*^*+/−*^ mice treated with AAV9-AntagoNAT-H via i.c.v. (dose A). We observed no statistical increase in *Scn1a* expression in the cortex and the heart (Figures S7A and S7B). VCN was significant in the cortex and heart compared to control groups (Figures S7C and S7D). Na_V_1.1 expression within the cerebral cortex remained unaltered, with no significant difference detected (Figure S7E).

As the construct contained a GFP reporter gene, we assessed GFP expression in the cortex and heart. We observed a significant increase of GFP in the cortex (Figure S6A). In the heart, we observed no significant GFP increase (Figure S6C). We observed no upregulation of CD68 (Figure S6E) and no significant changes VCN (Figure S6F) in the liver of AAV9-AntagoNAT-H *Scn1a*^*+/−*^ i.c.v. and i.v. group compared to PBS controls.

### P14 delivery of AAV9-AntagoNAT-H reduces SUDEP

**Figure 5:**
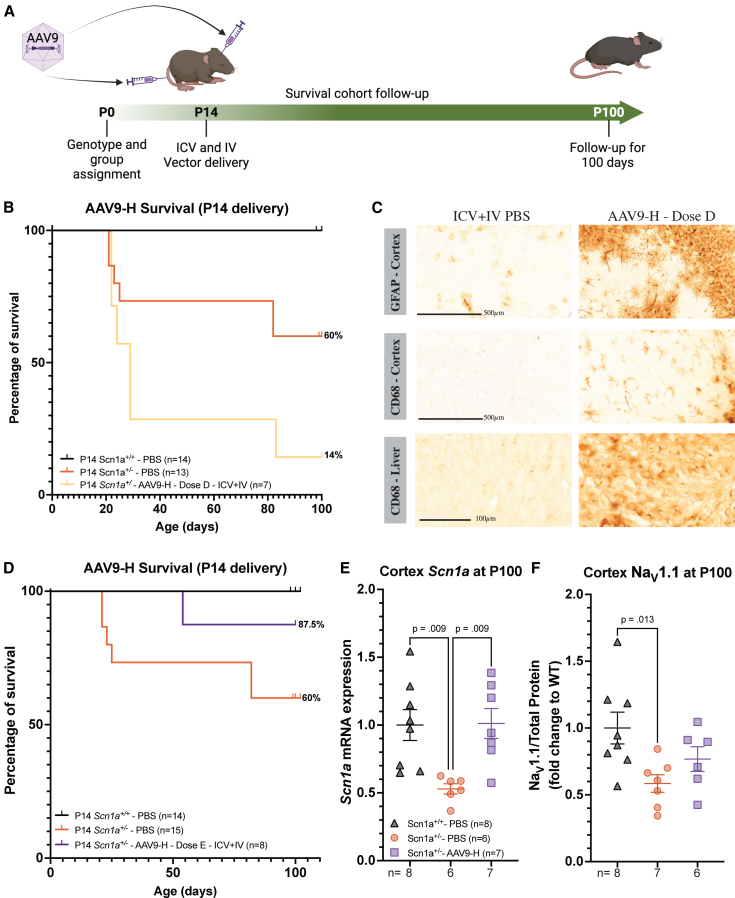
Increase in endogenous *Scn1a* after i.c.v. and i.v. AAV9-AntagoNAT-H therapy to P14 *Scn1a*^*+/−*^ DS mice (A) Schematic diagram showing the experimental plan. (B) Survival curve for AAV9-H dose D, showing percentage of survival. (C) Representative images of GFAP (top) and CD68 (middle) staining in the brain and CD68 in the liver (bottom) of animals that received AAV9-H dose D. Further details in Figure S9. (D) Survival curve of animals that received AAV9-H dose E, showing percentage of survival. Log-rank (Mantel-Cox) test. (E) *Scn1a* expression in cerebral cortex displayed a significant increase in both *Scn1a*^*+/+*^ PBS and *Scn1a*^*+/*^^−^ AAV9-AntagoNAT-H groups (*p* = 0.009) compared to *Scn1a*^*+/*^^−^ PBS group. Cerebral cortex tissues for *Scn1a*^*+/−*^ group, range from P20 to 100. (F) Na_V_1.1 expression in the cerebral cortex, reported a difference between control groups only (*p* = 0.013). One-way ANOVA Holm-Šídák’s multiple comparisons test.

The *Scn1a* transcript reaches a stable expression from P14 of development.^28^ We therefore asked whether delivering our AAV9-AntagoNAT-H treatment at P14, closer to the time point of seizure and SUDEP onset, could prolong survival in *Scn1a*^*+/−*^ DS model. We thus delivered AAV9-AntagoNAT-H to P14 *Scn1a*^*+/−*^ mice in a blinded and randomized study (Figure 5A). P14 *Scn1a*^*+/−*^ mice initially received the same dose as in the neonatal study (dose D: i.c.v.; 5 × 10^9^ vg per hemisphere and i.v.; 2.5 × 10^10^ vg, for a total dose of 3.5 × 10^10^ vg/mouse; Table S1). This dose was not efficacious, as the treated AAV9-AntagoNAT-H *Scn1a*^*+/−*^ mice yielded an overall survival of 14% compared to 60% of PBS control mice (Figure 5B). We noted upregulation of astrocytes and microglia in the brain and elevated macrophages in the liver of these treated *Scn1a*^*+/−*^ mice (Figures 5C and S9).

We, therefore, administered a lower dose. The P14 *Scn1a*^*+/−*^ mice received 2.5 × 10^9^ vg per hemisphere, via bilateral i.c.v. and 5 × 10^9^ vg via tail vein injection (dose E: total dose of 1 × 10^10^ vg/mouse; Table S1) and were monitored up to P100 (Figure 5A). We observed an 87.5% survival after gene therapy compared to 60% after PBS (Figure 5D). There was no difference in weight (Figure S8B). We repeated injections of dose E in P14 *Scn1a*^*+/−*^ mice and assessed febrile seizures between P20 and P25. However, we were unable to detect febrile induced seizures in treated and control groups (Figure S10).

*Scn1a* mRNA expression was measured by quantitative PCR (qPCR) of cerebral cortex of P100 mice and we observed a significant increase in endogenous *Scn1a* in treated group compared to PBS control group (Figure 5E). We were unable to detect *Scn1a* in the heart at this age. Furthermore, AAV9-AntagoNAT-H *Scn1a*^*+/−*^ group showed no significant increase in Na_V_1.1 expression in the cerebral cortex compared to PBS control group (Figure 5F).

Combination delivery of AAV9-AntagoNAT-H to P14 *Scn1a*^*+/−*^ mice resulted in GFP expression from the pre-frontal cortex to midbrain (Figures S11A and S11B) and GFP was quantified in the heart and revealed significant increase in GFP compared to PBS controls (Figure S12B). VCN analysis showed the vector was present in the cortex (Figure S11C) and heart (Figure S12A) at 100 days of development compared to PBS control group. We also observed no signs of elevated macrophages in the liver; however, a significant VCN (Figures S12C and S12D), compared to PBS control group was observed.

## Discussion

The AAV9-AntagoNAT-H therapy delivered to neonatal *Scn1a*^*+/−*^ mice via combination administration (i.c.v. and i.v.) significantly improves the disease phenotype in the *Scn1a*^*+/−*^ DS mice; reduces SUDEP (Figure 2B), susceptibility to febrile seizures (Figures 2D–2F), and seizure frequencies as assessed via EEG recordings (Figures 3B–3D). In addition, we measured a significant increase in endogenous *Scn1a* mRNA in the cortex (Figure 4A) of our treatment group compared to controls. We demonstrated an increase in GFP expression in the heart and no significant increase in VCN in the liver. AAV9-AntagoNAT-H was administered to juvenile *Scn1a*^*+/−*^ mice (P14), at a time point when *Scn1a* transcript expression levels are stable^28^; this revealed an increase in endogenous *Scn1a* mRNA. Our findings build upon previous work in which AntagoNATs were delivered via repeated intrathecal administration to a knockin DS mouse model, resulting in significant increase in *Scn1a* and improvements in seizure phenotype.^33^ By designing optimized sequences and incorporating them into an AAV9 viral vector, thereby providing a one-off treatment. More importantly, due to the broad biodistribution of AAV9 viral vectors^37^ and mechanism of action of the AntagoNATs,^33^ which should only have an effect in cells which express the *Scn1a* mRNA,^33^ we have validated a therapy that is able to achieve an extensive transduction profile after single administration (via i.c.v. and i.v.) and potentially target all cells expressing *Scn1a*. Therefore, we have not restricted our gene therapy to GABAergic inhibitory interneurons, which has been previously demonstrated in other gene therapy and editing preclinical studies for DS.^31^^,^^38^

In this study, we aimed to target the brain and heart by dual administration (bilateral i.c.v. and i.v.) of AAV9-AntagoNAT-H therapy. As previous studies have detected Na_V_1.1 expression in inhibitory interneurons,^7^ Purkinje neurons,^11^ CA1 pyramidal cells,^12^ and the heart,^16^ we firstly conducted a biodistribution study in the brain and heart of *Scn1a*^*+/+*^ mice administered with AAV9-AntagoNAT-H therapy by combination therapy and compared to i.c.v. group (dose A and B). i.c.v. Dose A group revealed a significantly greater GFP positive cells and VCN in the brain and heart compared to i.c.v. an i.v. dose C group. This was expected as the total VCN delivered to the i.c.v. group (dose A) was higher (1 × 10^11^ vg) compared to the combined approach (dose C; 3.5 × 10^10^ vg). However, when we administered dose A (i.c.v.) for AAV9-AntagoNAT-H, AAV9-AntagoNAT-K, and AAV9- CUR-1901 to *Scn1a*^*+/−*^ mice, we observed no significant increase in survival and a hyperactivity phenotype in treated groups compared to PBS controls. Although, hyperactivity has been noted in *Scn1a*^*+/−*^ mice in previous studies,^39^^,^^40^ we did not detect this phenotype in our PBS *Scn1a*^*+/−*^ mice, instead, it emerged specifically in the i.c.v.-treated groups at 1 × 10^11^vg. At P20, we assessed *Scn1a*, VCN, and Na_V_1.1 expression in *Scn1a*^*+/−*^ mice, which received dose A of AAV9-AntagoNAT-H via i.c.v. We detected no *Scn1a* change in the cerebral cortex and heart. Despite this, VCN was significantly high in the cortex and heart. Na_V_1.1 expression in the cortex showed no significant modulation following treatment. Thus, high dose A i.c.v. delivery did not modify survival, *Scn1a* gene expression, or Na_V_1.1 but substantially increase VCN in the cortical and heart tissue and was associated with robust hyperactivity phenotype. Additionally, we observed significant changes in the open-field assessment of mice that received i.c.v. high dose A in *Scn1a*^*+/−*^ for all three vector candidates (Figures S2A). As DS is a haploinsufficiency disease, genes are often dosage-stabilized, characterized by a narrow therapeutic expression window, where downregulation results in disease progression and over expression leads to toxicity.^41^ NATs are known to regulate their target genes through multiple mechanisms, including transcriptional interference, chromatin modulation, and post-transcriptional regulation.^42^ Importantly, increasing evidence indicates that NAT gene interactions can be dose and context-dependent, resulting in biphasic or non-linear effects on target gene expression.^42^ We therefore propose that at higher doses, excessive antisense activity may shift the regulatory balance, leading instead to suppression of *Scn1a* expression. We, therefore, hypothesize that high dose A i.c.v. delivery alone used in this study exceeds the optimal therapeutic range, resulting in toxic side effects rather than benefit, and that achieving efficacy will require careful dose optimization within a narrow therapeutic window. Although, we did not detect inflammation in the brain and the liver of *Scn1a*^*+/*+^-treated mice, to fully assess the safety profile of AAV9-AntagoNAT-H, we hypothesized that the observed behavioral abnormalities may be driven by excessive vector or overexpression of the CMV-driven GFP reporter.

In the neonatal study, we achieved a significant improvement in survival and a reduction in SUDEP events in our AAV9-AntagoNAT-H i.c.v.- and i.v.-treated group. Further evidence of AAV9-AntagoNAT-H i.c.v. and i.v. efficacy was observed in the assessment of febrile seizures, which are also observed in DS patients.^1^ The AAV9-AntagoNAT-H-treated group showed a significant reduction of seizure events and a reduction in severity compared to PBS controls. Spontaneous seizure events were assessed by EEG recordings from P30-45 of development. We observed a reduction in seizure frequency in the AAV9-AntagoNAT-H-treated *Scn1a*^*+/−*^ mice compared to PBS controls. Our results corroborate previous preclinical studies.^28^^,^^31^ Although these results are encouraging, further studies are required to increase the statistical power of this outcome measure.

We also demonstrated a significant increase in endogenous *Scn1*a mRNA expression in the cerebral cortex. Interestingly, we did not achieve a significant increase in Na_V_1.1 expression in the cerebral cortex of treated mice and we were unable to detect Na_V_1.1 in the heart. This may be due to the known difficulties with Na_V_1.1 antibodies, which previous studies have addressed by using reporter tags surrounding the *SCN1A* gene, to identify the Na_V_1.1 expression in DS mice.^26^^,^^27^ Also, low levels of Na_V_1.1 have been reported in human and dog hearts^16^ and therefore, the Na_V_1.1 levels in a mouse heart is perhaps too low to detect. Future studies would require assessment of sodium current specifically from Na_V_1.1 in cardiomyocytes.^16^ The Na_V_1.1 protein results in the cerebral cortex contrast with previous data, in which a significant increase of Na_V_1.1 was detected in the brain after a single neonatal i.c.v. administration of AAV9^31^ or ASO^28^ therapy to *Scn1a*^*+/−*^ DS mice. Our data could also provide evidence that normalization of in Na_V_1.1 expression to wild-type levels may not be entirely required to have a therapeutic benefit.

As our construct contained a GFP reporter gene, we assessed GFP expression in the brain and in the heart in *Scn1a*^*+/+*^ AAV9-AntagoNAT-H i.c.v.- and i.v.-treated group. We detected a widespread GFP expression in the brain by i.c.v. and i.v. route (Figure S5E). We observed a significantly higher GFP expression in the heart (Figure S5H) in i.c.v.-only-injected mice. Greater VCN were observed in the cortex and heart of i.c.v. injected mice than on i.c.v. and i.v. group (Figures S5G and S5I). In addition, we detected VCN in the liver of i.c.v. and i.v. groups (Figure S5K); interestingly, we observed no significant increase in inflammation in the liver (Figure S5J).

When we assessed VCN at P20 (Figure 4C) and at P100 (Figure S6B) in AAV9-AntagoNAT-H-treated *Scn1a*^*+/−*^ mice, we observed a marked reduction in the number of vector genomes. A significant reduction in vector genomes over development in the brain has been previously noted in mice after AAV9 delivery.^43^ We hypothesize that this could be the result of immune-mediated elimination of the viral genomes. However, we will require further studies to assess the immune response to AAV vector genomes over the course of development.

We observed differences in the number of GFP-positive cells between *Scn1a*^+/+^ (Figure S5F) and *Scn1a*^+/−^ (Figure S6A) mice following i.c.v. and i.v. administration of AAV9-AntagoNAT-H (dose C). This pattern was consistent with the VCN detected in these groups, where *Scn1a*^+/+^ mice exhibited higher VCN in the cortex compared with *Scn1a*^+/−^ mice (Figures S5G and S6B). The biological basis for this genotype-dependent difference in cortical VCN remains unclear. Further studies will be required to determine whether differences in vector uptake, distribution, or persistence contribute to this observation.

In this study, we aimed to deliver our AAV9-AntagoNAT-H therapy to older P14 *Scn1a*^*+/−*^ DS mice as the *Scn1a* transcript in the brain is stable at this time point.^28^ In addition, the rodent brain development at P14 is comparable to brain development of a 1–2 years old human brain.^44^ Our initial dose used at P14 (dose D) resulted in unexpected side effects, with reduced survival (Figure 5B) and signs of inflammation (Figures 5C and S9) in the brain and liver of treated mice. Treatment at P14 with a reduced dose (dose E) showed a significant increase in *Scn1a*; however, no significant changes in Na_V_1.1 compared to control mice (Figure 5F). By decreasing the total dose from 3.5 × 10^10^ vg to 1 × 10^10^ vg/mouse, we showed that this treatment has a narrow therapeutic range. Narrow therapeutic ranges have been demonstrated previously in pre-clinical AAV9 gene therapies for disease such as Spinal muscular atrophy.^45^ This is especially critical in the context of DS, where restoring Na_V_1.1 expression is crucial for maintaining neuronal excitability.^46^ As such, future studies will need to conduct a more refined dose-ranging evaluation of our AAV9-H therapy to determine the optimal therapeutic dose. We evaluated hyperthermia-induced febrile seizures between P20 and P25 in P14-treated *Scn1a*^*+/−*^ mice and did not observe seizure onset in either the AAV9-H or PBS groups (Figure S10). Because febrile-seizure susceptibility in *Scn1a* deficient mice is known to vary within a narrow developmental window,^47^ it is possible that this testing period did not coincide with peak vulnerability in our cohort. Future studies will therefore assess febrile seizures across multiple developmental stages to determine whether shifting the timing of AAV9-H delivery alters the age-dependent susceptibility profile. Furthermore, studies have noted that genetic mouse models are susceptible to genetic drifts, which can influence their phenotypes.^48^ We therefore consider genetic drift to be a potential contributing factor to the absence of febrile seizures observed between P20 and P25 in our study. Accordingly, future experiments will require rederivation or replenishment of the mouse strain to ensure genetic integrity and phenotypic consistency.

Nevertheless, this is the first study, to our knowledge, in which an AAV-based therapy has been delivered to P14 *Scn1a*^*+/−*^ DS mice. Further studies using an alternative heterozygous mouse model, where there is a longer period between first seizure and SUDEP and also other neurological symptoms such as cognitive and motor impairment,^27^ would be useful to further validate our treatment and to address the efficiency in older *Scn1a*^*+/−*^ DS mice after disease symptom onset.

We also assessed GFP expression profile in the brain and heart and VCN in the brain, heart, and liver. The GFP expression was observed from the pre-frontal cortex to midbrain (Figure S11A) showing a lower widespread expression profile than neonatal-treated mice. A significant GFP expression was detected in the heart. VCN assessment revealed a 10-fold increase in the cortex and a 4-fold increase in heart of P14-treated mice compared to neonatal. We detected similar VCN in the liver after bilateral i.c.v. and tail vein delivery (dose E) of AAV9-H to P14 *Scn1a*^*+/−*^ mice (Figure S12C) compared to neonatal i.c.v. and i.v. (dose C)-treated mice (Figure S6F). Studies have shown that the blood brain barrier (BBB) is functional by embryonic day 16^49^ and it’s permeability decreases over development, during the juvenile stage in rodents.^50^ This may explain the inability of AAV9 to cross the BBB in juvenile mice and the similar VCN in the liver, even though the dosage used was lower at P14. For future studies, we can explore lowering the vector dose for tail vein injections to P14 *Scn1a*^*+/−*^ mice. CMV promoters are widely used in gene therapy studies due to its strong and ubiquitous transcriptional activity. However, it is well documented that the CMV promoter is susceptible to progressive transcriptional silencing in the brain, primarily through epigenetic mechanisms such as DNA methylation.^51^ In our study, the AAV9 vector contained a CMV promoter driving GFP expression, which was employed for reporter gene analysis. We hypothesize that the observed reduction in GFP signal over time may be attributed to CMV promoter silencing. Consequently, future studies should consider incorporating alternative promoters with greater resistance to silencing, or alternatively, eliminating the CMV-GFP cassette altogether to improve long-term expression fidelity.

A recent study examined the expression profile of *SCN1A* long non-coding RNA (lncRNA) in brain samples from drug-resistant epilepsy patients.^52^ The study revealed that the *SCN1A* NAT is abundantly expressed across frontal and temporal lobe from brain samples between the age group of 1 and 19 years.^52^ This study validated the expression profile of *SCN1A* NAT in a human brain and therefore confirms the stable expression profile of our gene therapy target. Moreover, this supports and provides confidence for the applicability of the AAV9-AntagoNAT-H approach to be used in patients between 1– and 19 years.

There are a number of genetic therapeutic strategies developed for DS, including the AntagoNAT, which has shown decrease in seizure frequency after repeated intrathecal administration in a DS knockin model.^33^ Stoke Therapeutics have developed ASO therapy, which has shown a reduction of convulsive seizures in a phase 1/2a clinical trial after repeated intrathecal administration to DS patients between 2 and 18 years of age.^29^^,^^30^ The repeated administration of Zorevunersen resulted in 25% of patients exhibiting adverse event of post-lumbar puncture syndrome,^30^ which could present a barrier for long-term administration of this therapy. Encoded Therapeutics have developed an AAV9 transcription factor approach targeting GABAergic interneurons, where the preclinical study showed reduction of seizure phenotype and restoration of *Scn1a* and Na_V_1.1 expression after single neonatal i.c.v. delivery.^31^ Encoded Therapeutics has recently announced start date for phase 1/2 clinical trials.^32^ Our AAV9-AntagoNAT therapy, like Encoded Therapeutics’ approach, provides a one-off treatment; however, the added advantage of AAV9-AntagoNAT is that it is able to target additional cells in the CNS, which also express Na_V_1.1 protein. The addition of i.v. delivery of our AAV9-AntagoNAT therapy was to achieve efficient targeting of the heart, as the heart has shown to contribute to SUDEP through altered electrical function.^13^^,^^14^ However, we were unable to show sufficient increase of *Scn1a* expression and unable to detect Na_V_1.1 expression in the neonatal and P14 administration studies. Further studies, examining the electrical function in the heart after gene therapy would be useful to address the association with SUDEP.

Future studies would require RNA-seq to assess any off-target effects of the AntagoNATs employed in this study, increasing the number of animals injected for both neonatal and P14 *Scn1a*^*+/−*^ mice and conducting a dosage study to assess further effects on survival and seizure readouts. Furthermore, to evaluate the benefits of i.v. vector delivery in P14 *Scn1a*^+^/^−^ mice, a separate cohort of animals receiving i.c.v. administration of AAV9-H would be required for comparison. These additional experiments would be performed with a new version of the vector, without GFP. By using this approach, we would be able to establish therapeutic window in which, the vector does not show unwanted side effects.

Overall, we provide proof of concept preclinical evidence that neonatal i.c.v. and i.v. administration of AAV9-AntagoNAT-H, designed to specifically target *Scn1a* and *SCN1A* lncRNA can effectively restore *Scn1a* gene expression, modestly increasing Na_V_1.1 production, and reducing SUDEP incidences and seizures in a clinically relevant *Scn1a*^*+/−*^ DS mouse model. Additionally, administration at P14 showed an increase in endogenous *Scn1a* expression. In summary, the AAV9-AntagoNAT strategy provides great promise as a genetic therapy for DS and requires further preclinical testing to evaluate the safety and efficiency of this therapy.

## Materials and methods

### Cloning

The mouse *Scn1a* NAT secondary structure was predicted using Mfold.^53^ We mapped previously published AntagoNAT sequences^33^ to the resulting secondary structure files, and designed 18 new sequences targeting different locations of the NAT. These sequences were incorporated into an AAV backbone using gBlocks obtained from Integrated DNA Technologies (IDT, Europe) and cloned using InFusion cloning (Takara Bio, Europe). For the *in vitro* study, RNA polymerase II promoter, CMV was placed upstream of reporter gene, enhanced GFP, with mir-155 sequences flanking the AntagoNAT sequences to allow stable expression^34^; AAV-CMV-eGFP-mir-155-AntagoNAT-mir155-WPRE (Figure 1A). For the *in vivo* study, AntagoNAT sequences were driven by an RNA polymerase III promoter, U6. The construct also contained an additional CMV promoter to allow the expression of reporter gene, eGFP; AAV-U6-AntagoNAT-CMV-eGFP-WPRE (Figure 1B).

### *In vitro* cell assays

Around 30,000 N2a cells were seeded per well in 24-well plates using differentiation media: DMEM (Thermo Fisher Scientific) supplemented with 2% fetal bovine serum (FCS) (Sigma-Aldrich), 0.5 mM cyclic AMP (cAMP) (Merck) and 20 μM Retinoic Acid (Merck). At day 6, media was removed, and cells were transfected with plasmid (2 μg) containing candidate AntagoNAT sequence using Lipofectamine 2000 (Thermo Fisher Scientific) in OptiMEM (Thermo Fisher Scientific) overnight. Next day, the transfection media was removed and replaced with fresh differentiation media. For transduction experiments, media was removed from the cells and replaced with OptiMEM with a multiplicity of infection (MOI) of 1 × 10^6^ vector genomes per seeded cell. The cells were maintained for a further 5 days, at which point, they were collected using TRIzol reagent (Thermo Fisher Scientific). RNA was extracted as described in the following sections; gene expression was determined by qPCR.

### AAV vector production

Recombinant AAV vector was produced using a protocol previously described.^54^ AAVpro 293 T cells (Cat. No. 632273, Takara Bio) were transfected with AAV plasmid carrying the AntagoNAT sequence, an AAV2 Rep, and AAV9 Cap gene plasmid (University of Pennsylvania, USA) and adenovirus helper plasmid (Harvard University, USA) using polyethylenimine (PEImax, Polysciences Inc). The vector was purified in an AKTA prime plus HPLC machine (AKTA prime) using POROS CaptureSelect AAVX Resin (Thermo Fisher Scientific). The resulting AAV was treated with DNase I and then titrated by qPCR (in the following section). Vector stock was normalized to 1 × 10^13^ vector genomes/mL.

### Animals

All procedures were performed in accordance with the UK Home Office Animals (Scientific Procedures) Act 1986. All mice were housed under a non-reversed 12:12 h light-dark cycle and had access to food and water *ad libitum*. Animals were housed in individually ventilated cages with access to environmental enrichment. 129 S-*Scn1a*^tm1Kea^/Mmjax DS mouse strain, which contain an exon 1 deletion of *Scn1a*, resulting in haploinsufficiency, were obtained from the Jackson Laboratory (Jackson Laboratory, ME, USA). The heterozygous mice were crossed with wild-type C57BL/6 J mice (Charles River, UK). The resulting first generation (F1) of heterozygous mice was used for treatment assessment (F1; 129Sv × C57BL/6 J, from here onward, called *Scn1a*^*+/−*^ in the text*)*.^19^^,^^28^ Our humane endpoints were: weight loss of more than 15%, observation of two seizures and/or clear signs of illness (piloerection, hunched posture and labored breathing).

### Neonatal and P14 injections

At postnatal day 0/1 (P0/1) pups received combination bilateral i.c.v. and i.v. delivery. Bilateral i.c.v. administration was performed, where 5 μL of AAV9, or Dulbecco’s (PBS, Thermo Fisher Scientific) was administered to each hemisphere using a 33-gauge hamilton needle (VWR), following the previously described coordinates.^55^ The i.v. injection was performed by delivering 25 μL of AAV9 vector, or PBS to the superficial temporal vein using a 33-gauge needle following the procedure previously described.^56^ P14 delivery was performed in mice anesthetized with isoflurane, and received bilateral i.c.v. injection with 10 μL of AAV9, or PBS following previous protocols.^28^ An i.v. tail vein injection, delivered 30 μL of AAV9 vector or PBS. Doses used for each age/route of administration are detailed in Table S1. After the procedure, the mice were returned to their dam. Weight and survival were monitored.

### Temperature-induced seizures

Temperature-induced seizure assessment was conducted on *Scn1a*^*+/−*^ mice at P20 in neonatal injected mice. The mice were placed in a heated chamber and video was recorded. Starting temperature was 37°C, with incremental increases of 0.5°C every 1 min, until 43.5°C. Seizures were recorded at the corresponding temperature and assigned a Racine score.^57^ The measured temperature corresponded to the ambient temperature in the chamber. For P14 mice, the same protocol was followed but the procedure was performed between P20 and 25. After febrile-induced seizures, the mice were placed in a PhenoTyper home cage and video-recorded for 4 days.

### EEG recordings

DS mice (weight >20 g) were anesthetized with isoflurane (induction 5%, surgery 1.5%) and placed in a stereotaxic frame (Kopf). They were injected subcutaneously (s.c.) with buprenorphine (0.03 mg/mL) and Metacam (0.15 mg/mL). A wireless electrocorticogram (ECoG) transmitter (Cat. No. A3048P2-AA-C37-D, single-channel transmitter, Open Source Instruments) was implanted s.c. and the electrode was placed over the somatosensory cortex. EEG signals (sampled at 256 Hz) were recorded for 15 consecutive days for each animal. Epileptiform activity was analyzed post hoc, while blinded to the treatment group, using Pyecog software. EEG recordings were manually assessed, defined as a pattern of repetitive spike discharges followed by a progressive evolution in spike amplitude with a distinct postictal depression phase.

### Tissue collection and stereoscopic microscopy

Mice were anesthetized with isoflurane, an incision in the right atrium was made followed by perfusion by PBS to the left ventricle. Tissues were split in half, either stored in 4% paraformaldehyde (PFA) for 48 h, and then 30% sucrose at 4°C for immunohistochemistry, or snap frozen to −80°C for molecular analysis. Analysis of GFP expression using a stereoscopic fluorescence microscope (MZ16F; Leica, Wetzlar, Germany). Representative images were captured using a digital microscope camera (DFC420; Leica Microsystems, Milton Keynes, UK) and software (Image Analysis; Leica Microsystems).^58^

### Immunohistochemistry

Brain samples for immunohistochemistry were sectioned to 40 μm coronal sections using a sliding microtome (Carl Zeiss, Welwyn Garden City, UK), and stored at 4°C in a solution of 15% sucrose in Tris-buffered saline (TBS), 30% ethylene glycol, and 0.3% sodium azide. To visualize GFP an anti-GFP antibody (Abcam, ab290; 1:1,000) was used. For CD68, we used anti-CD68 antibody (BioRad, MCA1957; 1:200), GFAP was detected using an anti-GFAP Polyclonal antibody (Proteintech, 16825-1-AP; 1:1,000). Immunohistochemistry was performed following previously published protocols.^59^

Tissue images were captured using a stereoscopic fluorescence microscope (DM4000; Leica Microsystems (UK) Ltd) using Leica DFC7000 T camera (Leica Microsystems (UK) Ltd). Images were acquired using the Leica LAS X software v.3.4.2.18368 (Leica Microsystems (UK) Ltd). Quantification images were taken using a Leica HCX PL Fluotar 40×/0.75 na Objective (Leica Mikrosysteme Vertrieb GmbH, Germany). Variations in image background was corrected using Fiji (ImageJ2) v.2.14.0/1.54f,^60^ by subtracting a white field (empty) image from a quantification image. The script is available in supplemental materials. Quantitative image analysis was performed using Image-Pro Premier v.10 (Media Cybernetics). Quantification of fluorescent staining and cell co-localization was performed using CellProfiler v.4.2.8, following recommended protocol.^61^

### DNA and RNA isolation

DNA extraction for tissues was carried out using the DNeasy Blood and Tissue kit (QIAGEN) following manufacturer instructions. RNA extraction was performed using TRIzol reagent (Invitrogen) combined with the PureLink RNA Mini Kit (Invitrogen). For cells, media was removed, cells were washed with PBS, then 1 mL of TRIzol per 1 × 10^5^ to 1 × 10^7^ cells was applied. The lysate was triturated several times and transferred into a 1.5 mL tube. For snap frozen tissues, 1 mL of TRIzol was added per 50–100 mg. A 3 mm stainless steel bead (QIAGEN) was added and the tubes were placed on a TissueLyser II (QIAGEN, UK) and homogenized at 30 Hz for 3 min. The samples were processed immediately following the PureLink RNA Mini Kit instructions. Concentration was measured in a FLUOstar Omega microplate reader (BMG Labtech). Reverse-transcription was performed using the high-capacity cDNA Reverse Transcription kit (Applied Biosystems). The resulting cDNA was used for qPCR.

### qPCR

Primers and probes were designed to target the *Scn1a* and *Gapdh* genes. *Gapdh* was used as housekeeping. For *Scn1a;* forward: TCAGAGGGAAGCACAGTAGAC, reverse: TTCCACGCTGATTTGACAGCA, probe: CCAGAAGAAACCCTTGAGCCCGAA (fluorophore: ABY, quencher: QSY, sourced from Thermo Fisher Scientific). For GAPDH; forward: ACGGCAAATTCAACGGCAC, reverse: TAGTGGGGTCTCGCTCCTGG, probe: TTGTCATCAACGGGAAGCCCATCA (fluorophore: VIC, quencher: QSY, sourced from Thermo Fisher Scientific).

AAV vector titration was performed using primers targeting GFP; forward: GGCACAAGCTGGAGTACAAC, reverse: AGTTCACCTTGATGCCGTTC, probe: AGCCACAACGTCTATATCATGGCCG (fluorophore: FAM, quencher: ZEN/Iowa Black FQ, sourced from Integrated DNA Technologies, IDT). Standards for all genes of interest were sourced as gBlocks from IDT and a standard curve from 10^9^ to 10^3^ copies were used in all experiments. For titrations, the DNase I-treated vector was serially diluted 6 times and the average concentration from all dilutions within the standard curve was used as the vector titer. Luna Universal Probe qPCR Master Mix (NEB) with 250 nM of primers and probes was used in all reactions. About 5 μL of each cDNA/vector sample was used. The plate was run in a QuantStudio 3 instrument (Applied Biosystems). Data were analyzed using the QuantStudio Design and Analysis v.1.4 (Applied Biosystems) software. Technical replicates were assessed and accepted if they were between 0.5 CTs from each other. Two samples from the heart RNA assessment (Figure 4B) had to be discarded as they did not show gene expression of either *Scn1a* or *Gapdh*.

### Capillary immunoassay

Tissues were homogenized in T-PER Tissue Protein Extraction Reagent (Thermo Fisher Scientific) with 1X complete, EDTA-free Protease Inhibitor Cocktail (Merck) using a QIAGEN TissueLyser II (30 Hz for up to 2 min). Lysates were incubated on ice for 5 min then sonicated for a further 5 min. Lysates were centrifuged at 14,000× g at 4°C for 15 min after which supernatants containing the membrane-enriched fraction were centrifuged for a further 5 min. Concentrations of resulting protein lysates were determined using the BioRad DC protein assay (BioRad) according to manufacturer’s instructions. A capillary immunoassay using these protein lysates was performed on the ProteinSimple Jess System (Bio-techne) using the manufacturer’s template for a 66-440kDa RePlex chemiluminescence assay (Bio-techne) with total protein normalization. A total of 0.5 mg/mL lysates and anti-Na_V_1.1 antibody (Alomone ASC-001; 1:50) were used as previously described.^62^ Automatic normalization to total protein and quantification of Na_V_1.1 protein signal was achieved using Compass for Simple Western software (Bio-techne). Raw data for this assay are available in Table S2. Original files are available in the corresponding zip files.

### Statistical analysis

GraphPad Prism (v.10.5, Boston, MA, USA) was used for statistical analysis. For *in vitro* data, one-way ANOVA with a post hoc analysis using the two-stage linear step up procedure of Benjamini, Krieger, and Yekutieli multiple comparison. For smaller group number comparisons, we used the post hoc analysis using the Dunnett’s multiple comparison test. For comparison between specific groups, we used the Kruskal-Wallis test with Dunn’s multiple comparisons test. Power and sample size calculation program^63^ v.3.1.6 (October 19, 2018) was used to determine animal numbers for the gene therapy study. For *in vivo* assessment, the following statistical analysis were used: survival data was analyzed using the log-rank (Mantel-Cox) test, for weight, a mixed-effects model (REML) with a post hoc analysis using the Dunnett’s multiple comparisons test was performed. For EEG data, two-way ANOVA with the Greenhouse-Geisser correction was performed. To compare means between two groups from the EEG data, unpaired Mann-Whitney test was used. To assess DNA and RNA gene expression data we used one-way ANOVA with a post hoc analysis using the Holm-Šídák’s multiple comparisons test. Data presented as mean (standard deviation, SD). The *n* number for each experimental group is specified in the figures and figure legends. The *p* values are also shown in figures. Normal distribution of the data was assessed with the D'Agostino-Pearson’s test. Outliers were identified using the ROUT method available in GraphPad Prism.

## Data and code availability

The data that support the findings of this study are available from the corresponding author, upon request.

## Acknowledgments

10.13039/100012357LifeArc P2020-0008 and P2023-0011 (to R.K., J.A.D., E.M.C., S.N.W., S.S., A.M., G.L., and H.C.). Great Ormond Street Hospital Children Charity and Dravet Syndrome UK Charity V4720 and V4919 (to R.K., J.A.D., S.N.W., S.S., and E.C.). Therapeutic Acceleration Support (TAS), UCL (to R.K. and G.L.). GOSH/Spark Research grant V4019 (to G.L.). Medical Research Council Programme grant MR/V034758/1 (to G.L. and S.S.). Medical Research Council Development Pathway funding scheme MR/Z505201/1 (to R.K., E.M.C., S.S., A.M., G.L., and J.H.C.). Epilepsy Research UK Emerging Leader Fellowship F1701 (to A.B.). Medical Research Council New Investigator project grant MR/S011005/1 (to G.L.). Research conducted by A.M. and J.H.C. is supported by the National Institute for Health and care research Great Ormond Street Hospital Biomedical Research Centre (10.13039/501100000272NIHR GOSH BRC). A.M. received funding support from the 10.13039/501100000265MRC (MR/T007087/1), Great Ormond Street Hospital Children Charity (VS0122), Rosetrees Trust, and Wellcome Trust TIN Scheme. The graphical abstract and Figures 1A, 1C, 2A, 3A, 5A, and S10A were created with BioRender.com

## Author contributions

J.A.D., investigation, methodology, data curation, formal analysis, and manuscript writing; E.M.C. and A.A.B., investigation and formal analysis; A.K., S.G., V.P., Z.W., and M.K., investigation; A.M., J.H.C., and S.S., methodology; G.L., investigation and methodology; S.N.W., conceptualization; R.K., conceptualization, methodology, visualization, data curation, project administration, and writing – original draft.

## Declaration of interests

J.H.C. is president of the International League Against Epilepsy (2021–2025) and chair of the medical boards for Dravet UK, Hope 4 Hypothalamic Hamartoma and Matthew’s friends. S.S. is listed as inventors on patent WO2018229254A1. G.L. and S.S. have equity in a company that aims to bring epilepsy gene therapy to the clinic.

## Supplemental information

Data S1. Table S2

Document S2. Article plus supplemental information
