# Supplementary material for: AAV9-mediated targeting of natural antisense transcript as a novel treatment for Dravet syndrome
Source: Mol Ther Nucleic Acids. 2026 Apr 30;37(2):102942. doi: 10.1016/j.omtn.2026.102942 (PMC13196359; doi:10.1016/j.omtn.2026.102942)
Supplement: Document S1. Figures S1–S12 and Table S1 [file mmc1.pdf]

OMTN, Volume *37*

Supplemental information

AAV9-mediated targeting of natural antisense transcript as a novel treatment for Dravet syndrome

Juan Antinao Diaz, Ellie M. Chilcott, Amanda Almacellas Barbanoj, Anna Keegan, Sonam Gurung, Valda Pauzuolyte, Zak Waddington, Maria Kyriacou, Amy McTague, J Helen Cross, Stephanie Schorge, Gabriele Lignani, Simon N. Waddington, and Rajvinder Karda

Figure S1

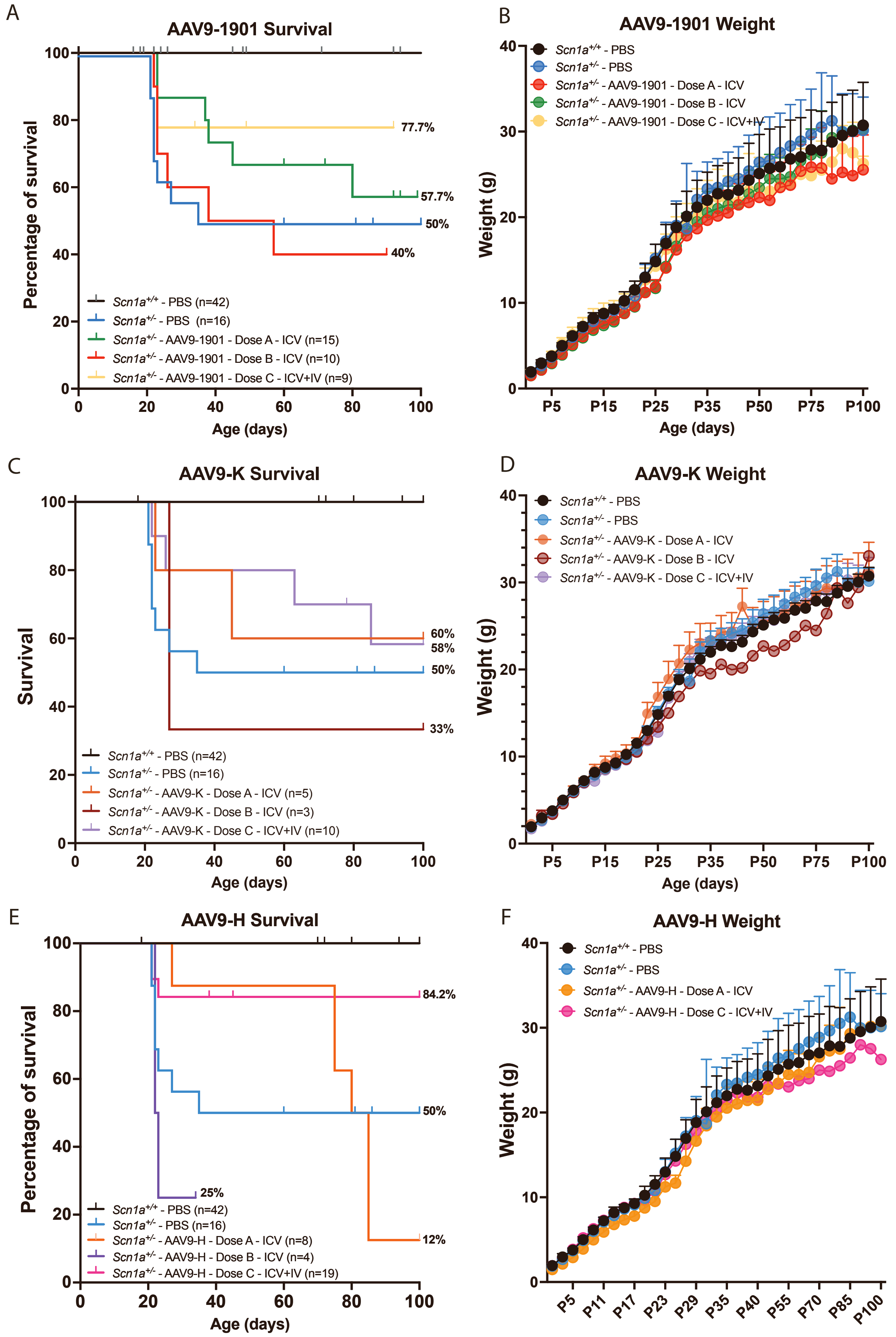

Figure S1 - Survival and weights of *Scn1a*$^{+/-}$ DS mice treated with AAV9-1901, AAV9-K and AAV9-H. (A) Survival curve of *Scn1a*$^{+/-}$ DS mice treated with AAV9-1901 (Comparted to *Scn1a*$^{+/-}$ PBS; Dose A: p=0.349, B: p=0.662, C: p=0.177) and (B) their corresponding weights. (C) Survival of AAV9-K treated *Scn1a*$^{+/-}$ mice (Comparted to *Scn1a*$^{+/-}$ PBS; Dose A: p=0.240, B: p=0.777, C: p=0.418) and (D) their corresponding weights. (E) Survival of AAV9-H treated *Scn1a*$^{+/-}$ mice (Comparted to *Scn1a*$^{+/-}$ PBS; Dose A: p=0.285, B: p=0.519, C: p=0.039) and (F) their corresponding weights, dose C shown for comparison, same data shown in Figure 2B and C. Log-rank (Mantel-Cox) test. Weights analysed by Two-Way ANOVA with Dunnett's multiple comparisons test.

Figure S2

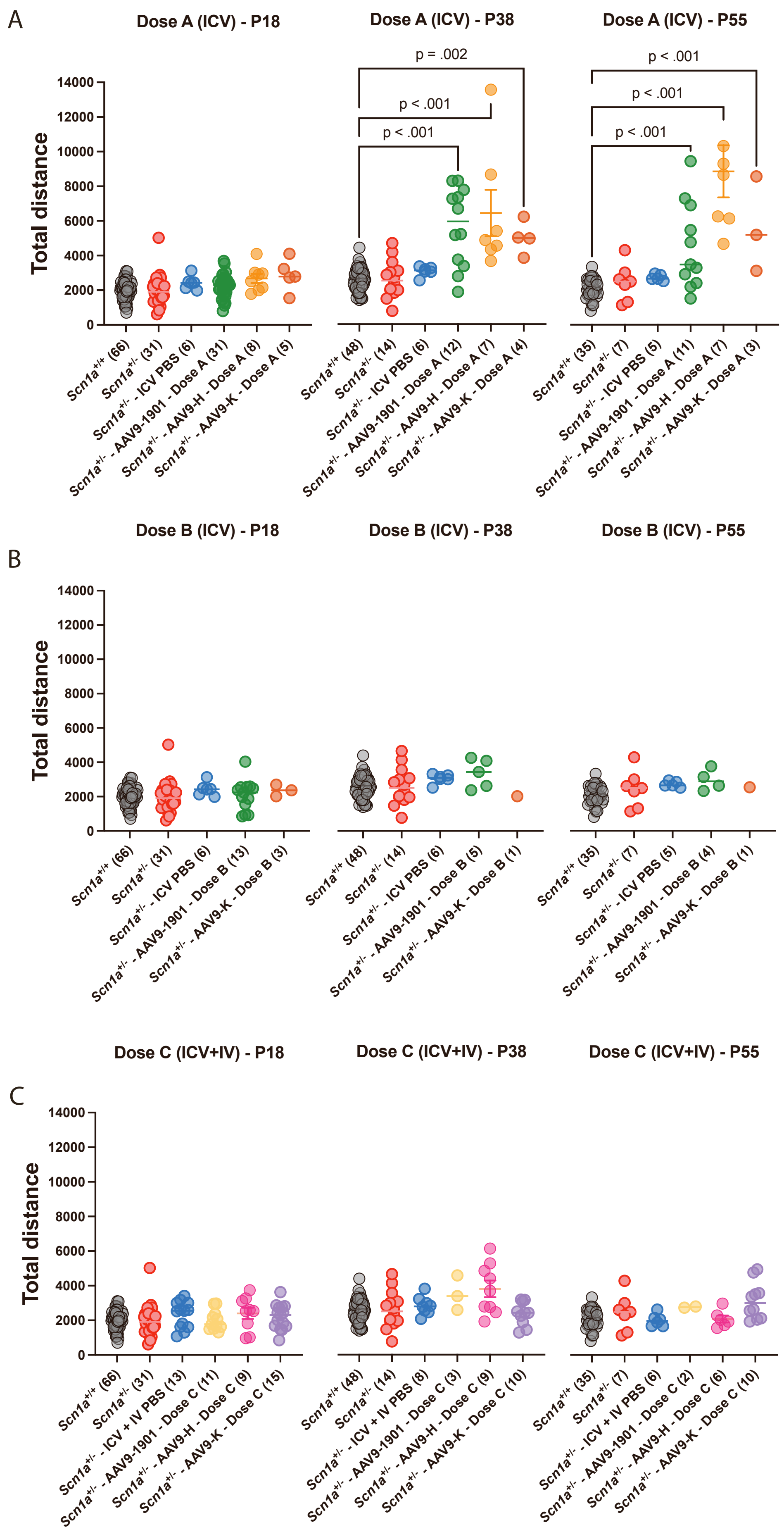

Figure S2 - Open-field assessment on treated *Scn1a*$^{+/-}$ mice. Open-field was assessed at three development ages; P18, P38 and P55. (A) There was a significant increase in total distance travelled in *Scn1a*$^{+/-}$ mice which received dose A (1x10^{11}vg) of AAV9-1901, AAV9-H and AAV9-K via ICV compared to *Scn1a*$^{+/+}$ controls. (B) No significant differences were observed with dose B (1x10^{10}vg). (C) *Scn1a*$^{+/-}$ mice treated with dose C (3.5x10^{10}vg) of AAV9-1901, AAV9-H and AAV9-K via ICV & IV showed no significant differences compared to *Scn1a*$^{+/+}$ littermates. Analysed by One-Way ANOVA with Holm-Šídák's multiple comparisons test. N numbers indicated in parenthesis for each group and age.

Figure S3

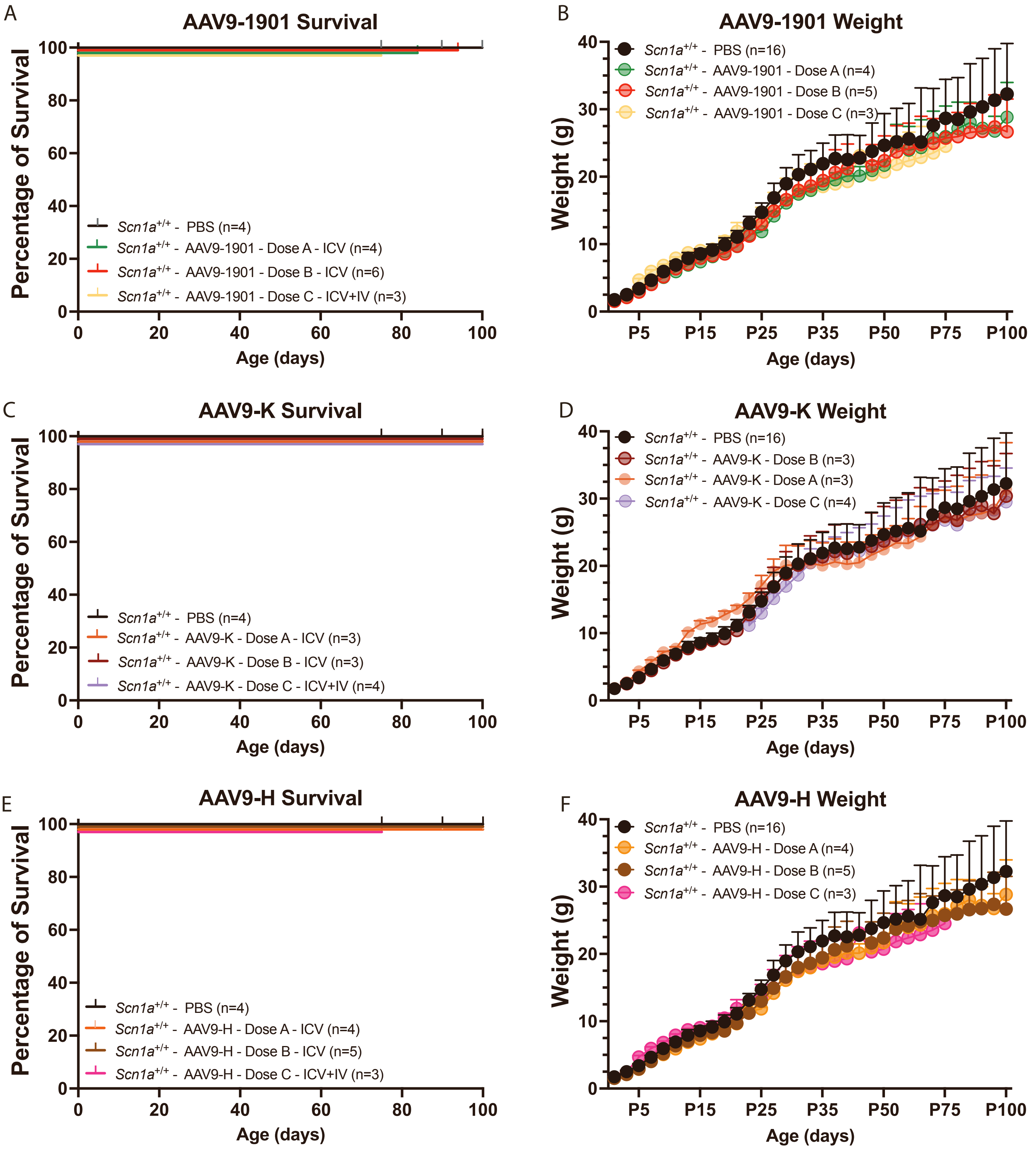

Figure S3 - Survival and weights of *Scn1a*$^{+/+}$ treated mice. (A) Survival curve of *Scn1a*$^{+/+}$ DS mice treated with AAV9-1901 and (B) their corresponding weights. (C) Survival of AAV9-K treated mice and (D) their corresponding weights. (E) Survival of AAV9-H treated mice and (F) their corresponding weights. Log-rank (Mantel-Cox) test. Weights analysed by Two-Way ANOVA with Dunnett's multiple comparisons test.

Figure S4

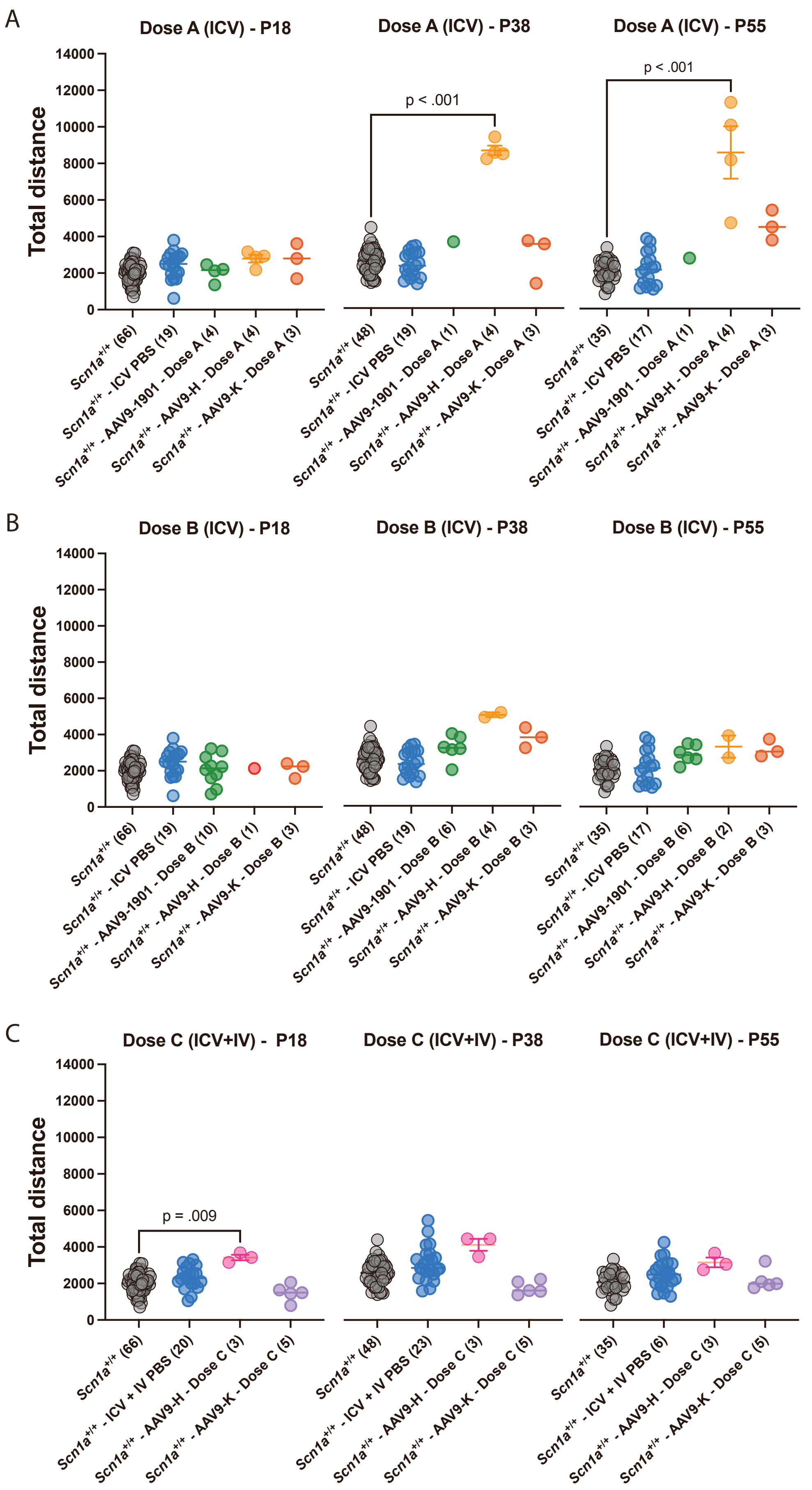

Figure S4 - Behavioural assessment of *Scn1a*$^{+/+}$ treated mice. Open-field was assessed at three ages: P18, P38 and P55. (A) At P38 and P55 AAV9-H treated mice (Dose A: 1x10^{11}vg) showed a significant increase in the total distance travelled. (B) No significant differences were observed in mice treated with dose B. (C) At P18 AAV9-H mice treated with dose C showed a significant increase in total distance travelled compared to control group. N numbers indicated in parenthesis for each group and age. Analysed by One-Way ANOVA with Holm-Šídák's multiple comparisons test.

Figure S5

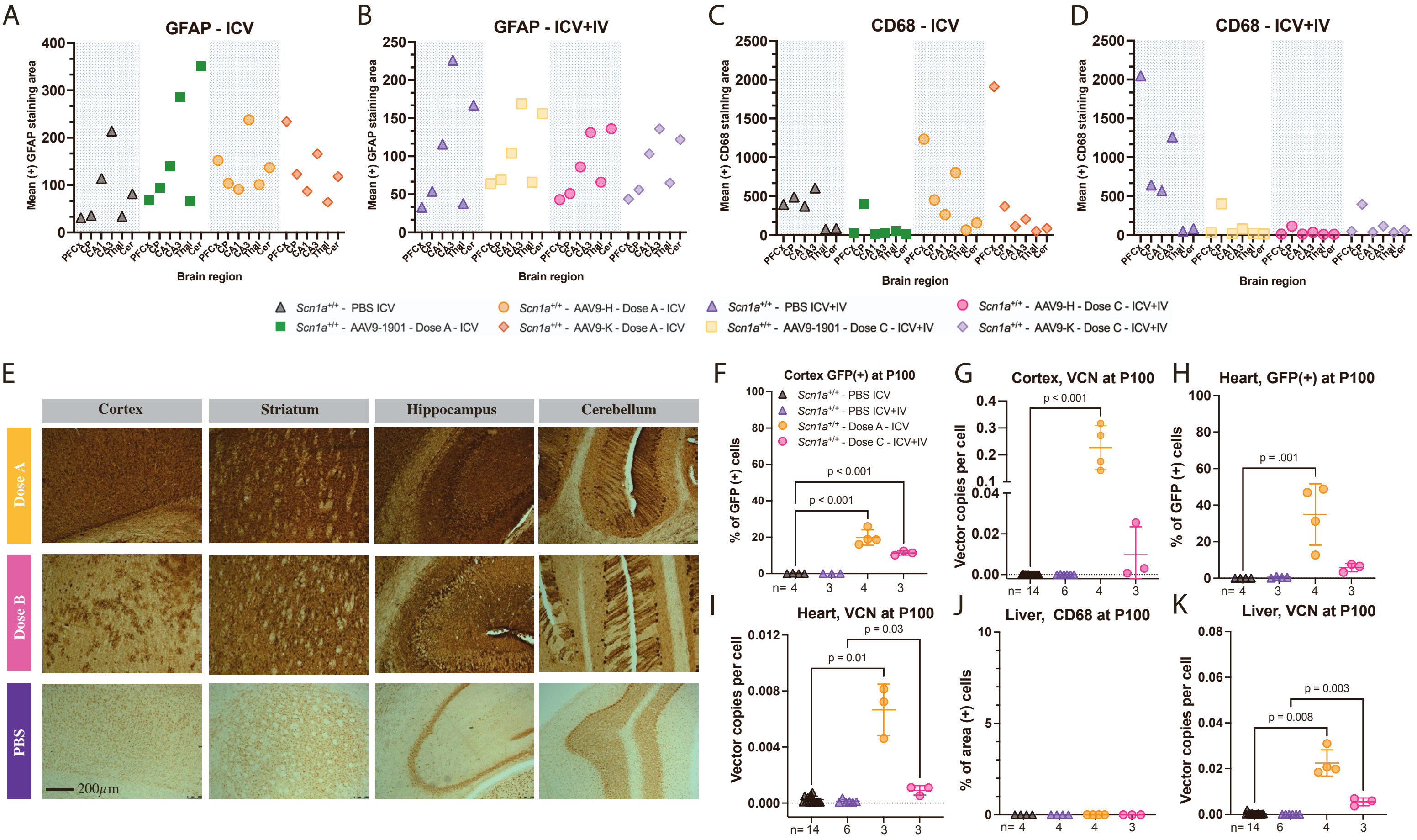

Figure S5 - Molecular assessment of *Scn1a*$^{+/+}$ treated mice. (A and B) Quantification of GFAP and (C and D) CD68 staining showed no significant differences between all treated groups compared to PBS controls. PFCx = Prefrontal cortex, CP = Caudoputamen, CA1 = CA1 region of the hippocampus, CA3 = CA3 region of the hippocampus, Thal = Thalamus, Cer = Cerebellum. Analysed by One-Way ANOVA with Dunnett's multiple comparisons test. n= 6 for each region. (E) Representative images of GFP staining in the brain. (F) Quantification of fluorescent staining against GFP in the cortex. ICV and ICV and IV delivery showed a significantly higher persentage of GFP positive cells compared to controls. (G) VCN of ICV treated mice showed a mean of 22 out of 100 cells with the vector. ICV and IV delivery showed a mean of 1 in 100 cells with the vector. (H) Quantification of fluorescent staining against GFP. ICV delivery showed a significantly higher percentage of GFP positive cells compared to controls in *Scn1a*$^{+/-}$ treated mice. (I) ICV and ICV and IV treated mice showed significant increase compared to control. (J) CD68 staining quantification in *Scn1a*$^{+/+}$ showed no significant differences between groups. (K) VCN in ICV treated mice showed that 2-5 per 100 cell had a vector copy. ICV and IV treated mice had every 3-5 per 1000 cells with a copy of the vector. Analysed with Kruskal-Wallis test with Dunn's multiple comparisons test.

Figure S6

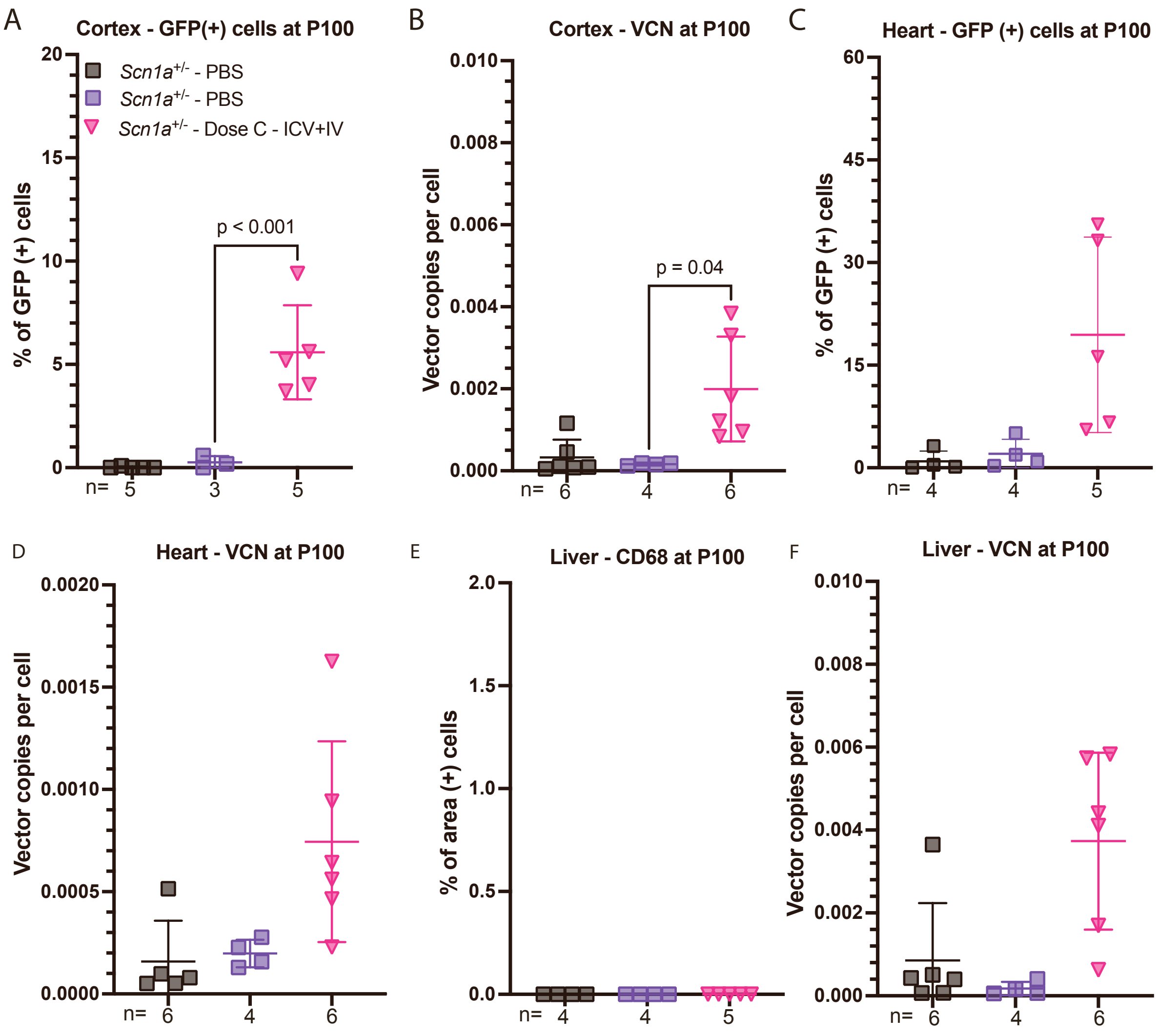

Figure S6 - GFP quantification and VCN in the cortex, heart and liver of *Scn1a*$^{+/-}$ treated mice. (A) Quantification of GFP (+) cells in the cortex of *Scn1a*$^{+/-}$ mice treated with AAV9-H at $3.5x10^{10}$vg (Dose C). (B) VCN in the cortex of *Scn1a*$^{+/-}$ mice; treated mice showed a mean of 2 vector genomes per 1000 cells. (C) Quantification of fluorescent staining against GFP in the heart of *Scn1a*$^{+/-}$ mice treated with AAV9-H (Dose C). (D) VCN assessment in the heart of treated mice. No significant difference were observed. *(*E) Quantification of CD68 staining in the liver of treated *Scn1a*$^{+/-}$ mice showed no significant differences between groups. (F) VCN assessment in liver tissue of treated *Scn1a*$^{+/-}$ showed 3-5 copies of the vector per 1000 cells, this did not reach significance. Analysed with Kruskal-Wallis test with Dunn's multiple comparisons test.

Figure S7

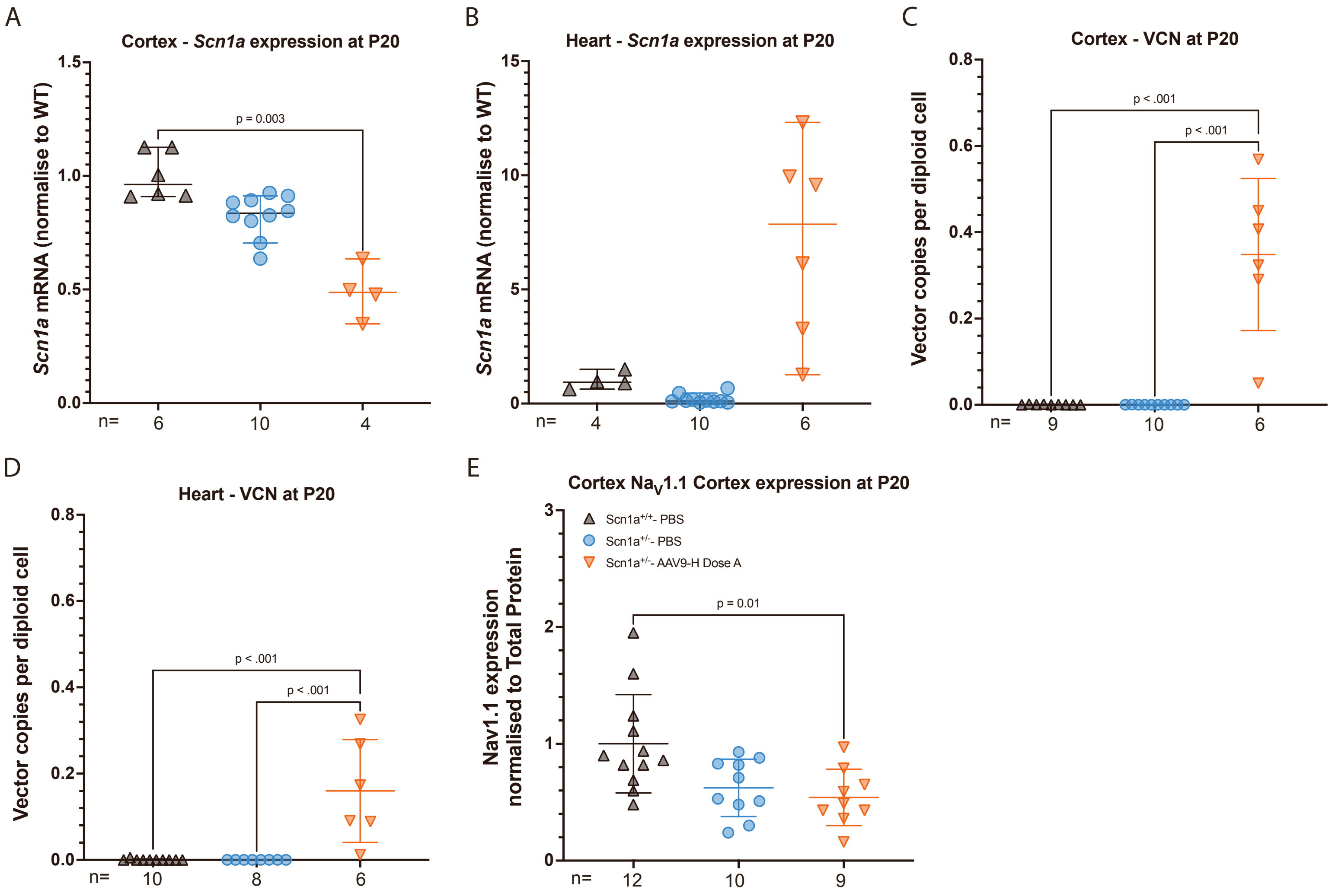

Figure S7 - Analysis of *Scn1a*, VCN and Na$_V$1.1 expression in AAV9-H ICV (Dose A) group at P20. (A) *Scn1a* expression in the cortex. (B) *Scn1a* expression in the heart. (C) Vector copy number analysis in the cortex. (D) Vector copy number in the heart. (E) Na$_V$1.1 expression in the cortex. One-Way ANOVA Dunn's multiple comparisons test. n numbers for each group are indicated in the figure.

Figure S8

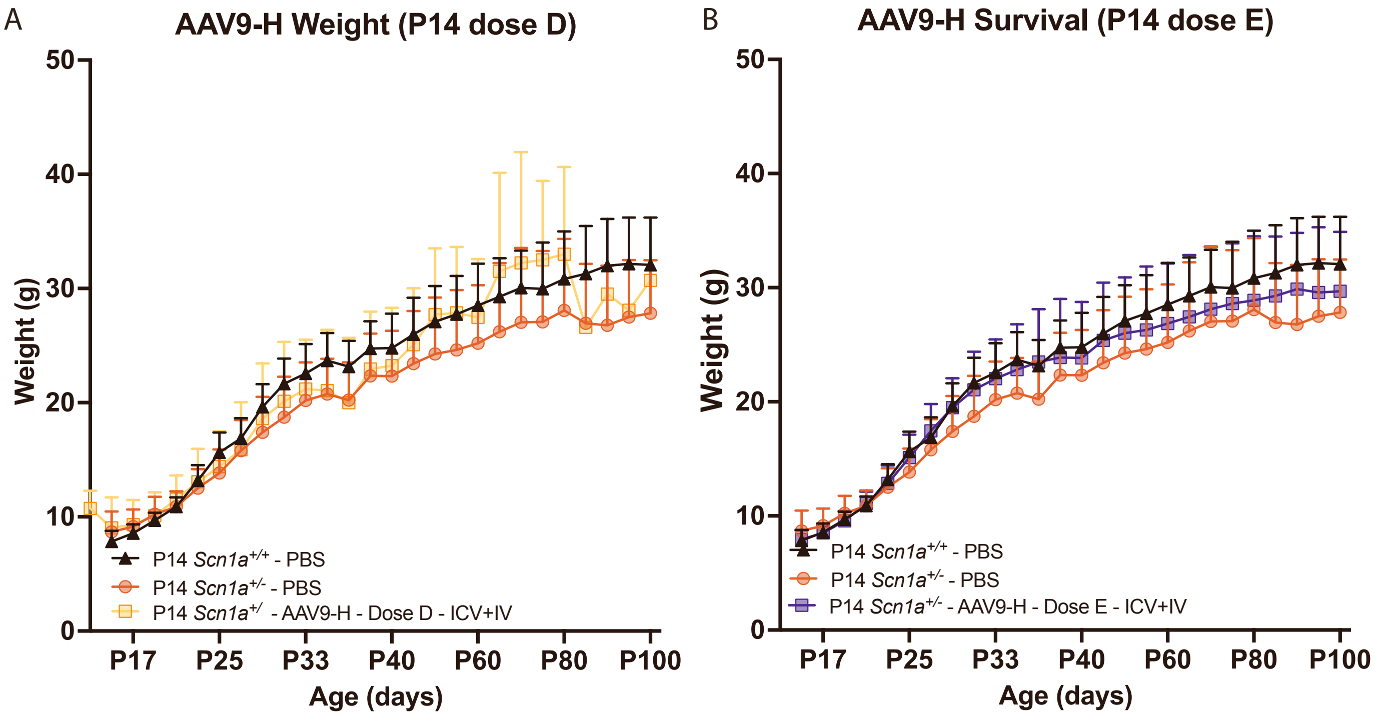

Figure S8 - Weights of P14 *Scn1a*$^{+/-}$ mice treated with AAV9-H - Dose D and E. (A) Weights of *Scn1a*$^{+/-}$ DS mice treated with AAV9-H dose D (3.5×10^{10}vg). (B) Weights of *Scn1a*$^{+/-}$ DS mice treated with AAV9-H dose E (1×10^{10}vg). Two-Way ANOVA with Dunnett's multiple comparisons test.

Figure S9

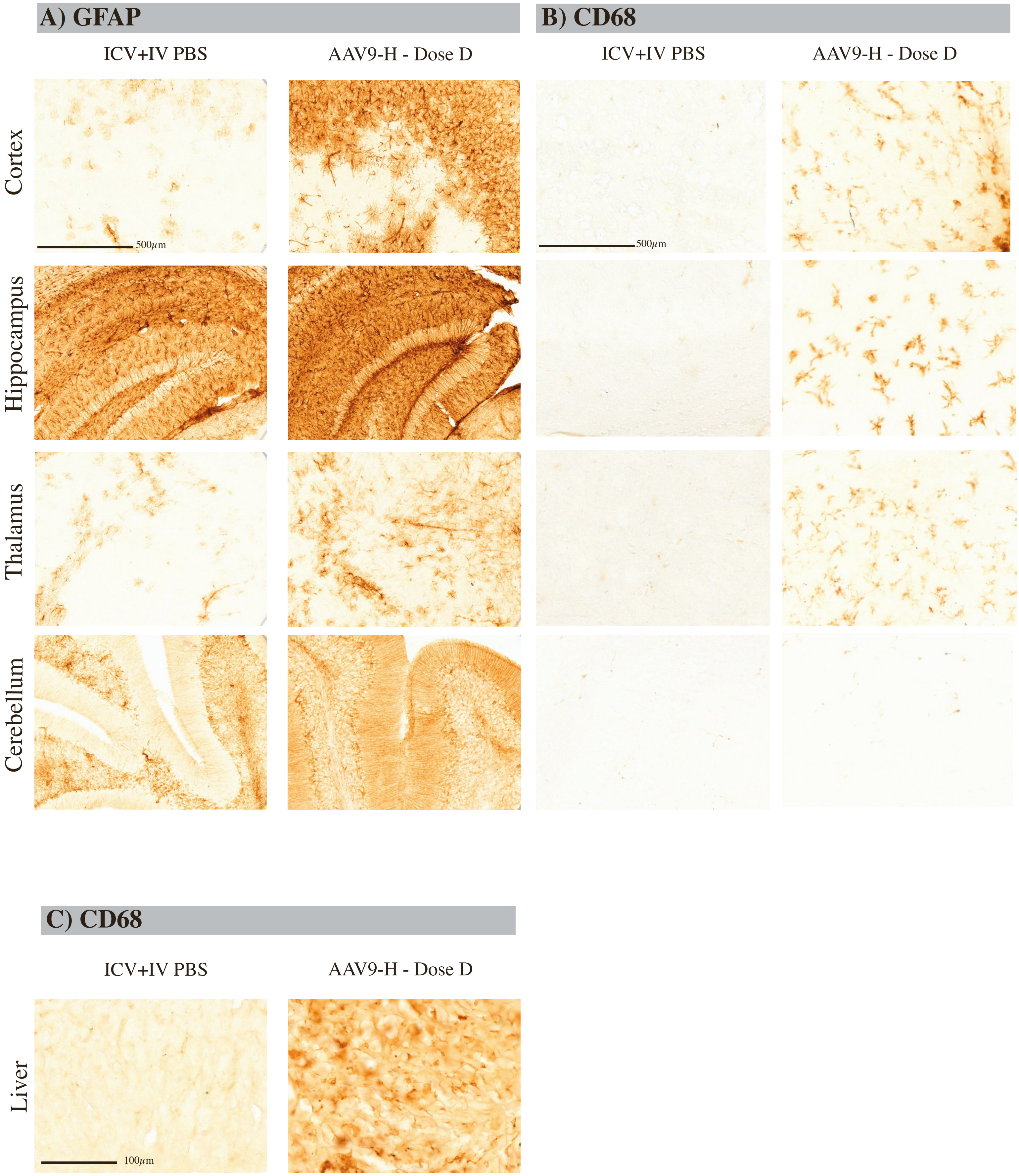

Figure S9 - Immunohistochemical analysis on brain and liver of P14 treated mice treated with AAV-9 Dose D. (A) Representative images of GFAP stain to detect astrocytes in the brain of AAV9-H treated at dose D ($3.5x10^{10}$vg/mouse) and PBS control group. (B) Representative images of CD68 stain to detect microglia in the brain of AAV9-AntagoNAT-H treated and PBS control group. (C) Representative images of macrophage activation in the liver of treated mice. Images from the cortex (GFAP and CD68) and liver (CD68) were reused from Figure 5C.

Figure S10

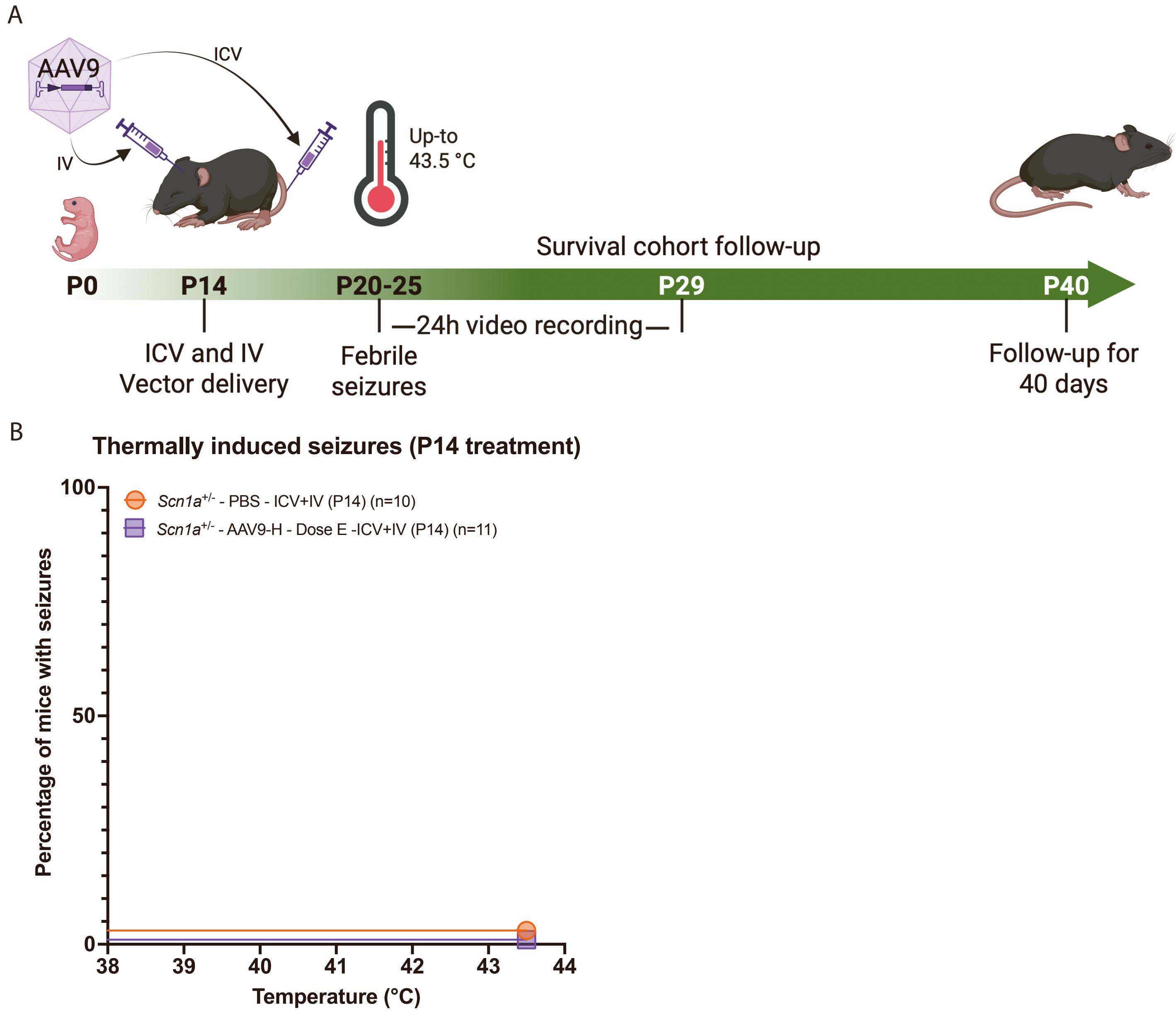

Figure S10 - Febrile seizures in DS mice treated with AAV9-H delivered via ICV+IV injection (Dose E) at P14. (A) Experiment timeline. (B) Febrile seizure temperature threshold assessment. Log-rank (Mantel-Cox) test.

Figure S11

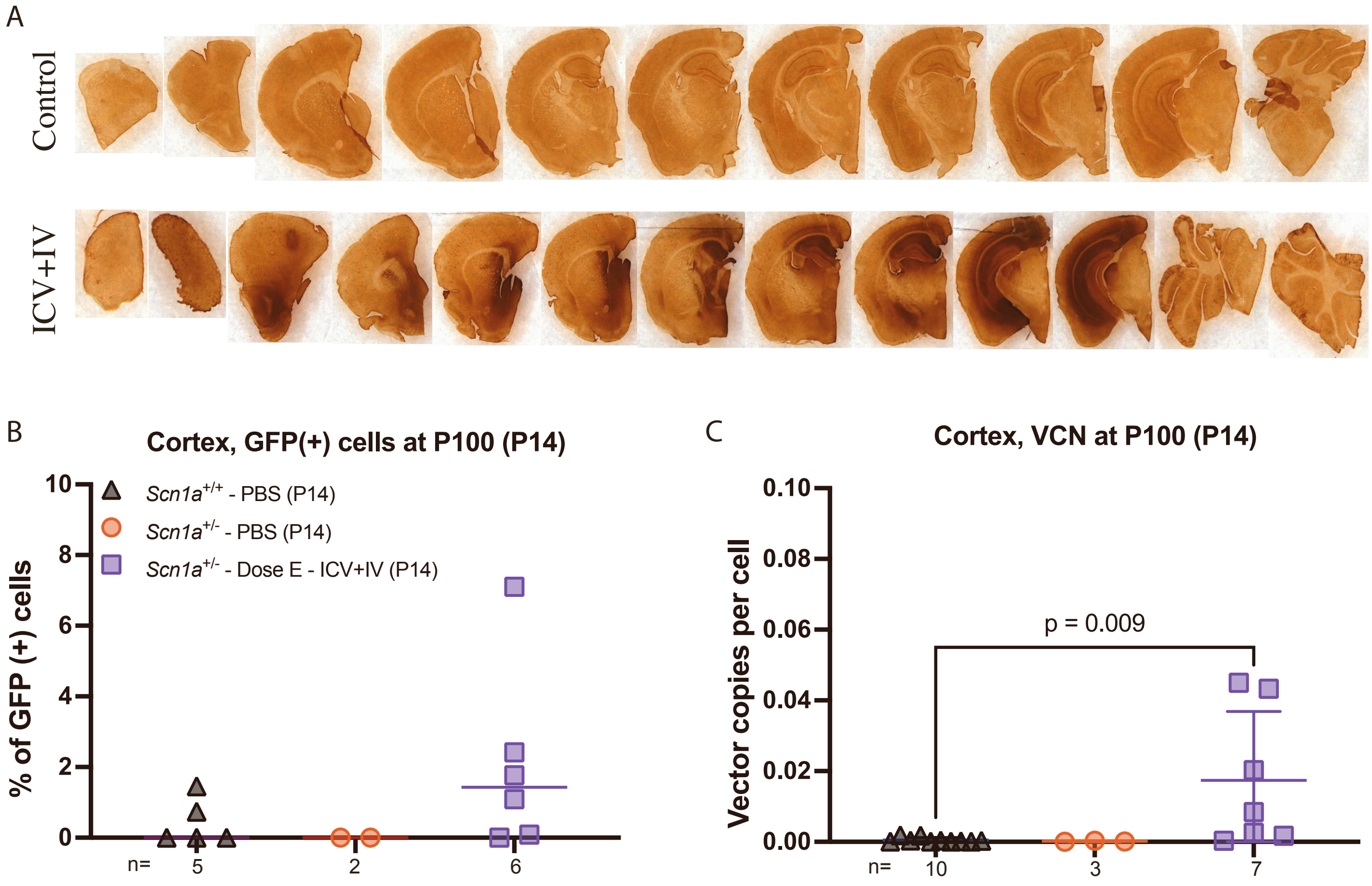

Figure S11 - Biodistribution of AAV9 vector delivered via ICV+IV at P14. (A) *Scn1a*$^{+/+}$ mice received AAV9-H at P14 *via* ICV and IV (Dose E; 1x10^{10}vg/mouse, 5x10^{9} vg per route). At 100 days of development, brain tissues were harvested for immunohistochemical analysis. Representative images. (B) Quantification of GFP expression in the cortex. We observed no significant differences. (C) Assessment of the number of vector copies of AAV9-H in the cortex of P14 treated mice, we found an average of 2 out of 100 cells had the vector. Analysed with Kruskal-Wallis test with Dunn's multiple comparisons test.

Figure S12

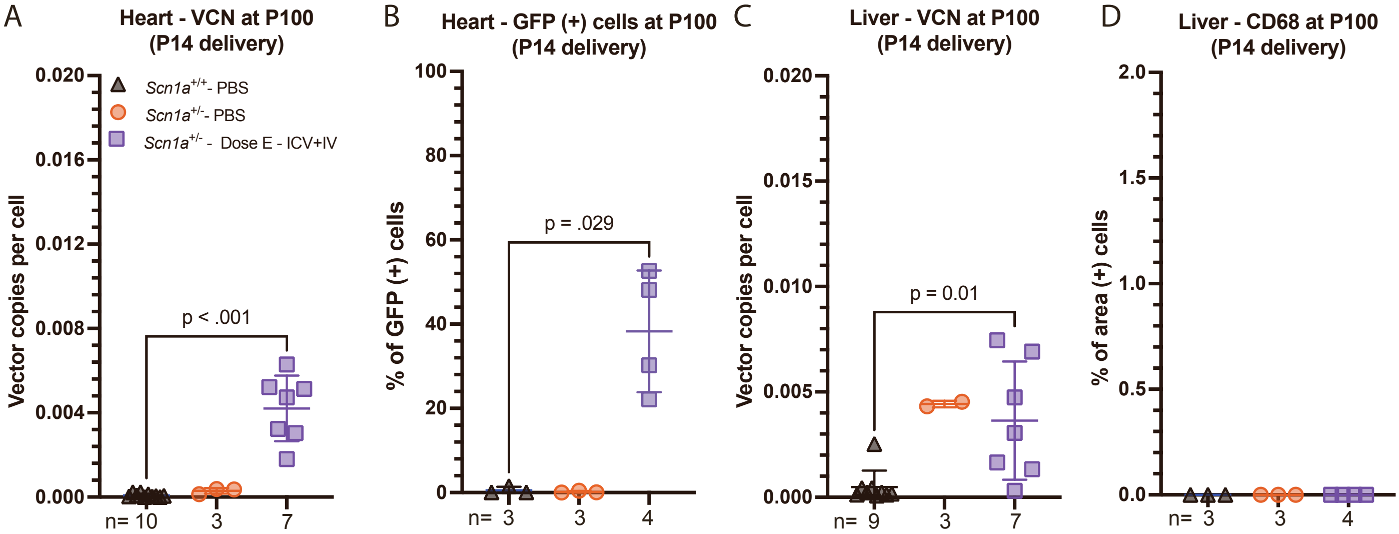

Figure S12 - Immunohistochemistry and VCN assessment of mice treated at P14 with AAV9-H (Dose E; $1x10^{10}$vg/mouse). (A) VCN in the heart of P14-treated mice collected at P100, which showed around 4 in 1000 cells had a vector genome. (B) Percentage of positive GFP cells in the heart of P14 treated mice collected at P100, around 38% of cells were found to be GFP positive. (C) Assessment of the number of vector copies of AAV9-H in the liver of treated mice, an average of 3 out of 1000 cells had the vector. (D) Quantification of CD68 staining the liver of treated mice showed no significant differences between groups. Analysed with Kruskal-Wallis test with Dunn's multiple comparisons test.

Supplemental Tables

Table S1: AAV9 vector doses used in the study. N/A = route not used. P0/1 = Post-natal day 0/1, P14 = Post-natal day 14.

Dose Label	ICV dose, per hemisphere	IV dose	Total dose, per mouse	Age of delivery
**A**	$5x10^{10}$vg	N/A	$1x10^{11}$vg	P0/1
**B**	$5x10^{9}$vg	N/A	$1x10^{10}$vg	P0/1
**C**	$5x10^{9}$vg	$2.5x10^{10}$vg	$3.5x10^{10}$vg	P0/1
**D**	$5x10^{9}$vg	$2.5x10^{10}$vg	$3.5x10^{10}$vg	P14
**E**	$2.5x10^{9}$vg	$5x10^{9}$vg	$1x10^{10}$vg	P14

Table S2: Raw values from the capillary immunoassay for figures 4E, 5F and S7E. Files from the immunoassay software are available in the .zip files for each of the corresponding figures.

Supplemental Videos

Video S1: Febrile seizure recording from *Scn1a*$^{+/-}$ mouse.

Video S2: Febrile seizure recording from AAV9-H treated *Scn1a*$^{+/-}$ mouse.
